# Supplementary figures and images for: NANOG is repurposed after implantation to repress Sox2 and begin pluripotency extinction
Source: EMBO J. 2025 Aug 18;44(19):5337–74. doi: 10.1038/s44318-025-00527-9 (PMC12488938; doi:10.1038/s44318-025-00527-9)

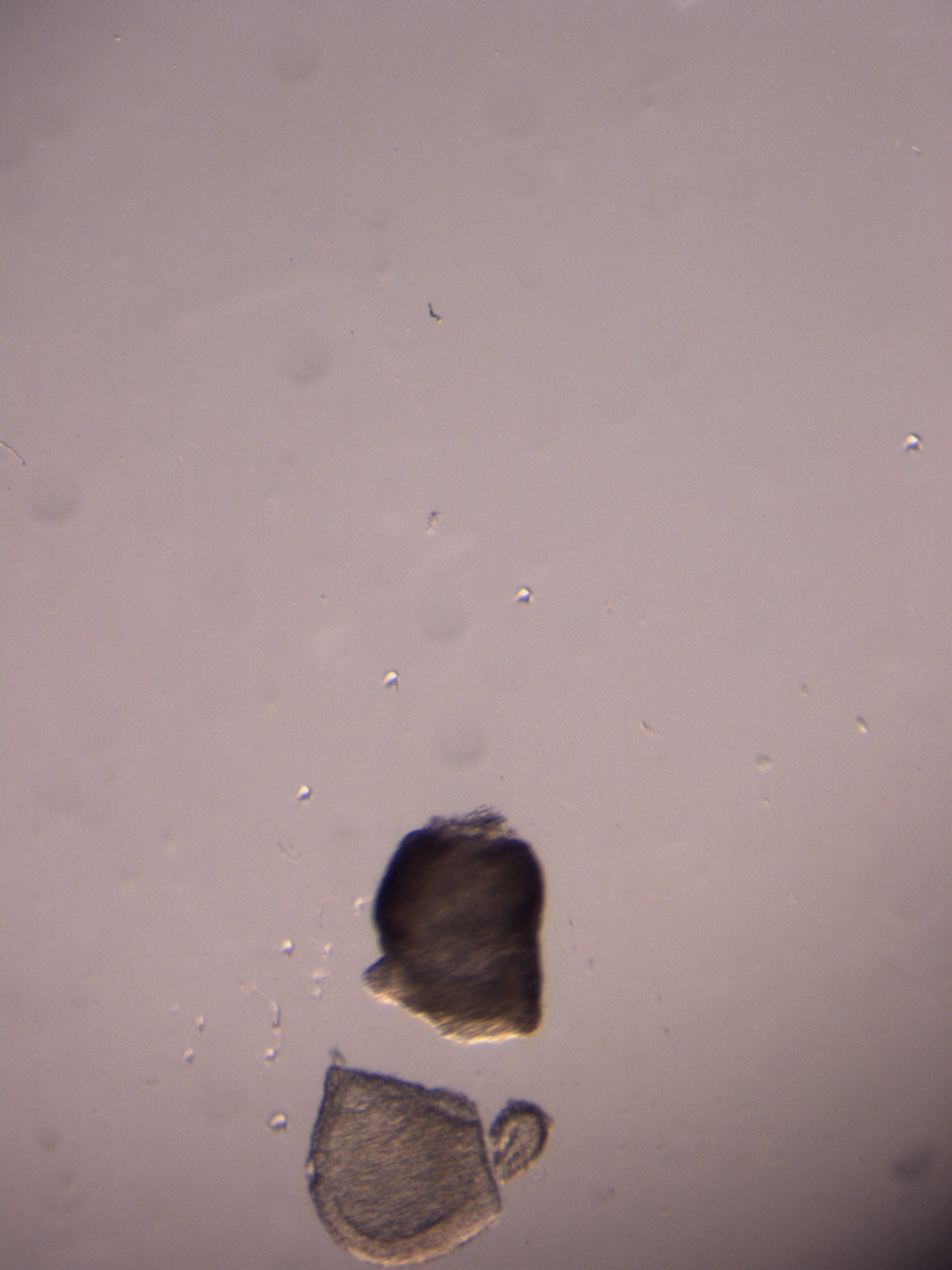

Supplement: Supplementary file 6 — Source data Fig. 2 [file 44318_2025_527_MOESM6_ESM.zip › Figure 2/2B/Embryo Region 1 cut.tif]

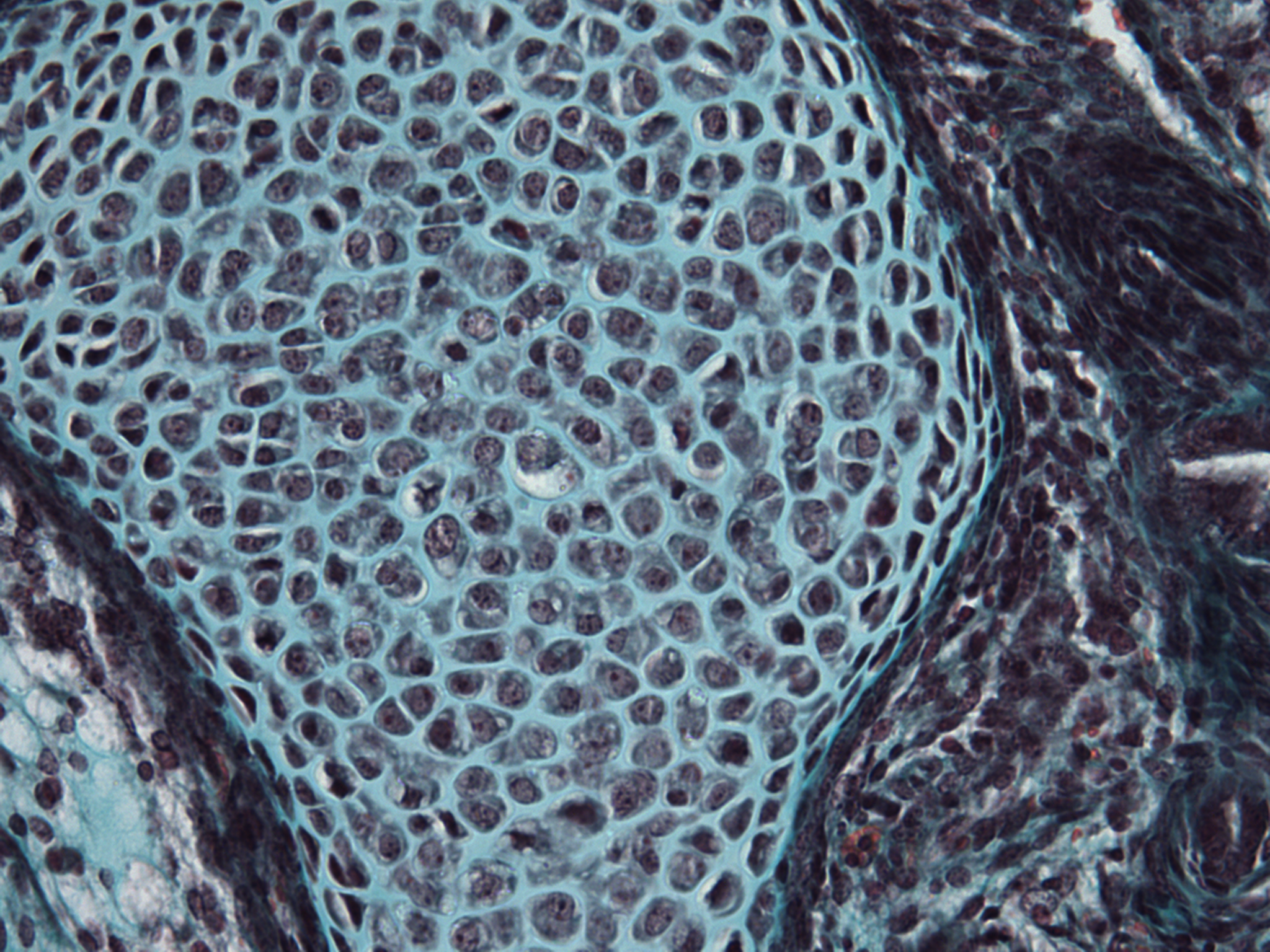

Supplement: Supplementary file 6 — Source data Fig. 2 [file 44318_2025_527_MOESM6_ESM.zip › Figure 2/2C/Region R - Chondrocyte (Mesoderm).tif]

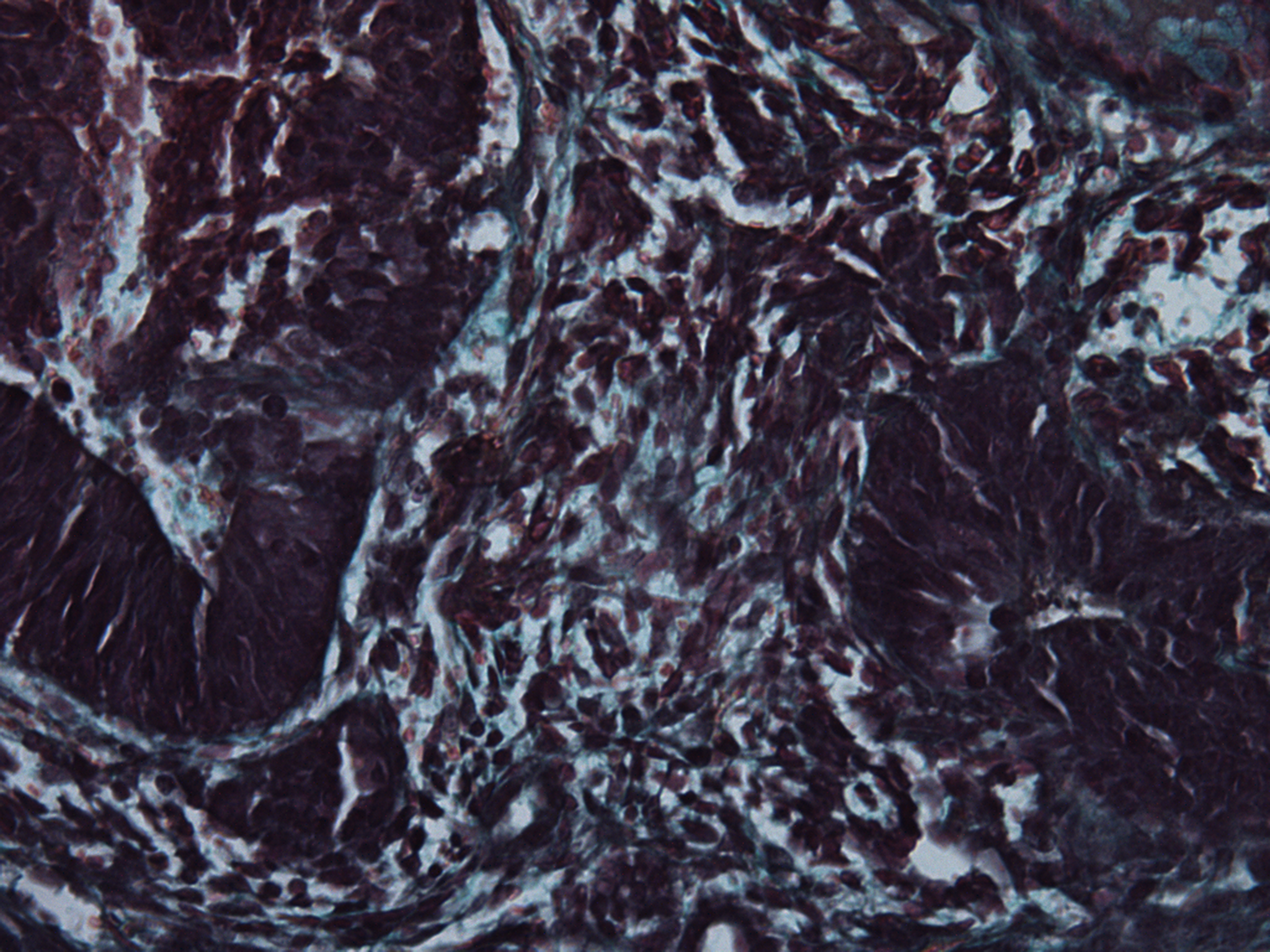

Supplement: Supplementary file 6 — Source data Fig. 2 [file 44318_2025_527_MOESM6_ESM.zip › Figure 2/2C/Region R - Embryonal Carcinoma.tif]

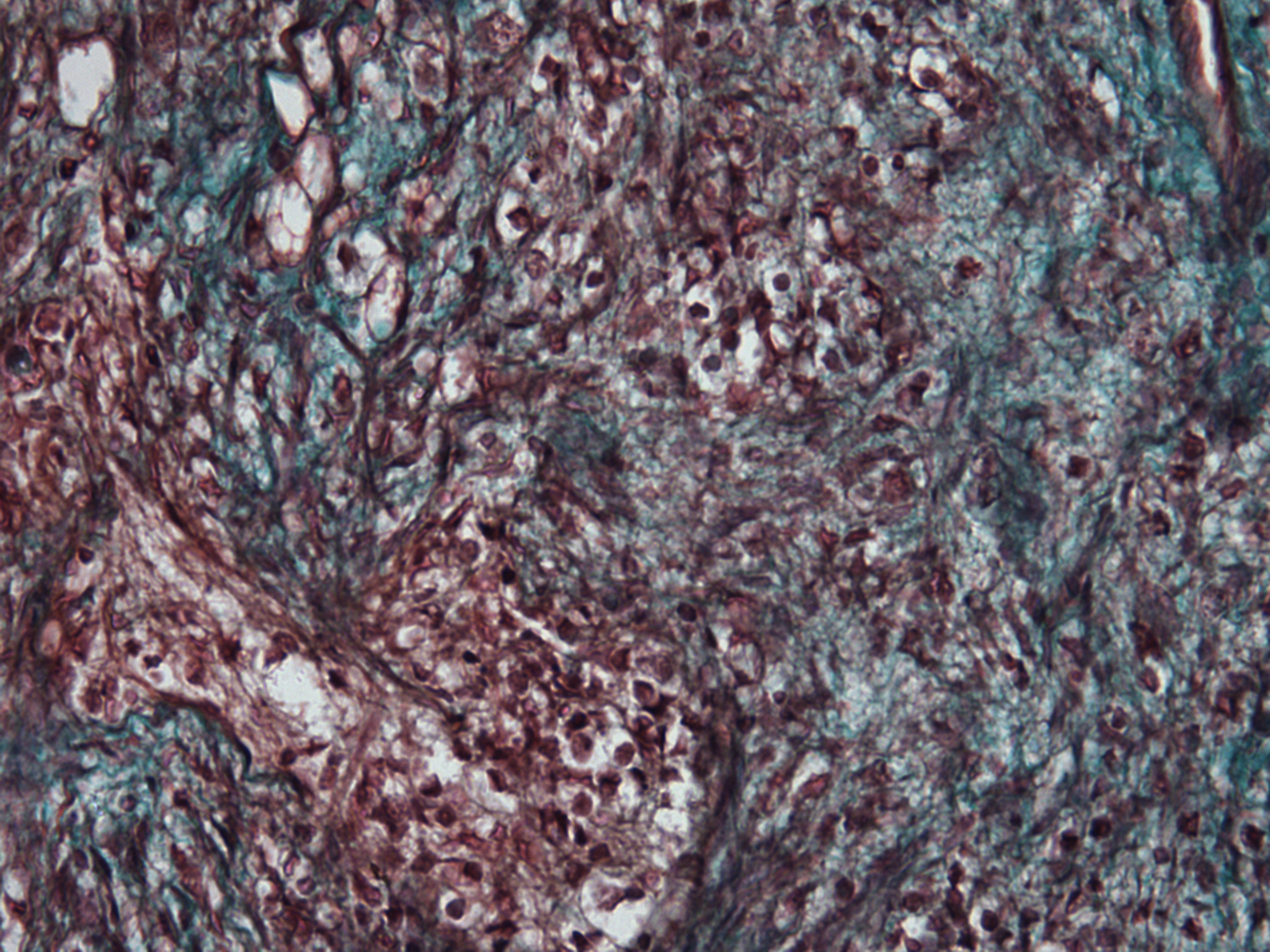

Supplement: Supplementary file 6 — Source data Fig. 2 [file 44318_2025_527_MOESM6_ESM.zip › Figure 2/2C/Region R - Nerve Tissue (Ectoderm).tif]

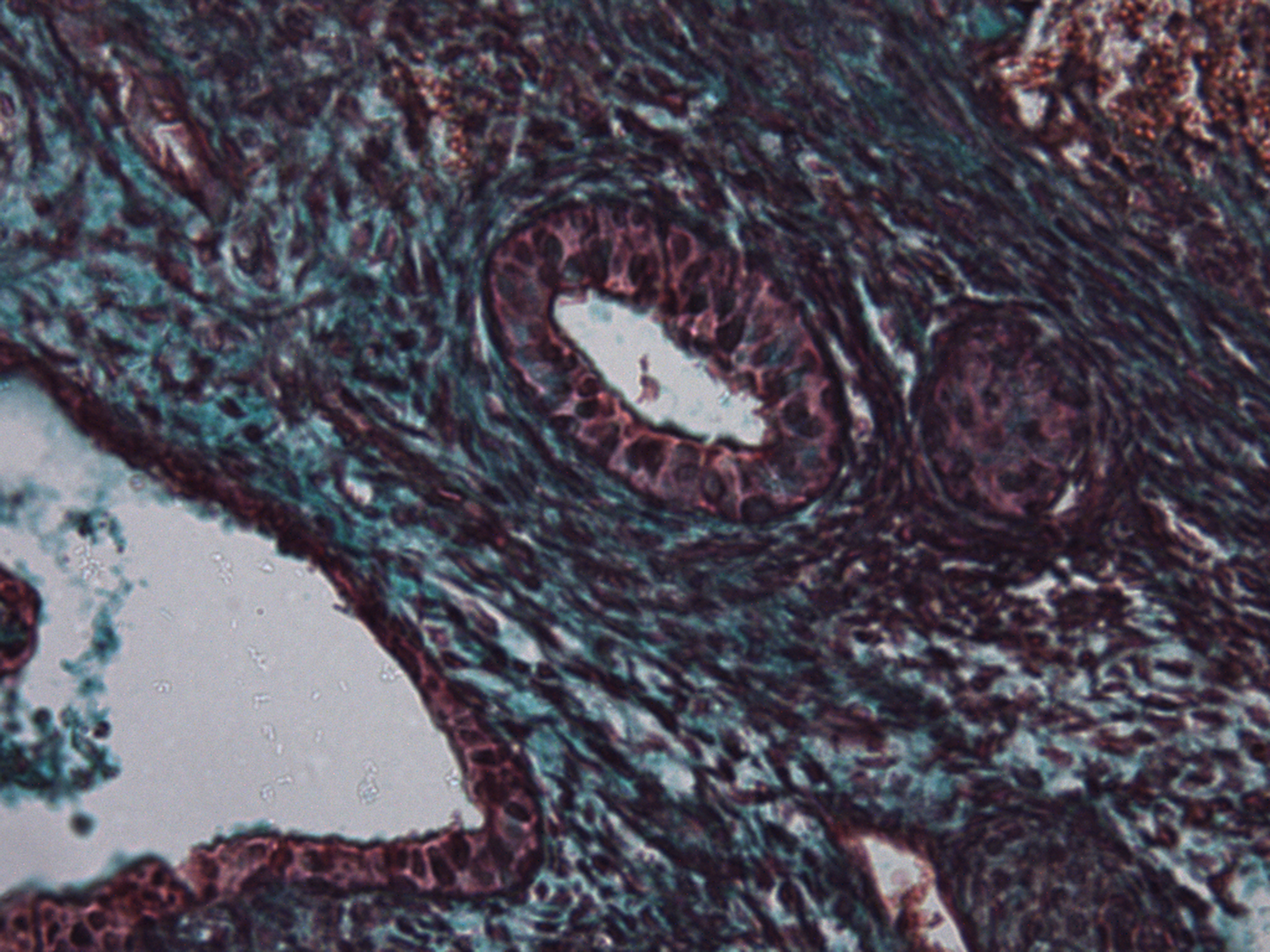

Supplement: Supplementary file 6 — Source data Fig. 2 [file 44318_2025_527_MOESM6_ESM.zip › Figure 2/2C/Region R - Respiratory Epithelium (Endoderm).tif]

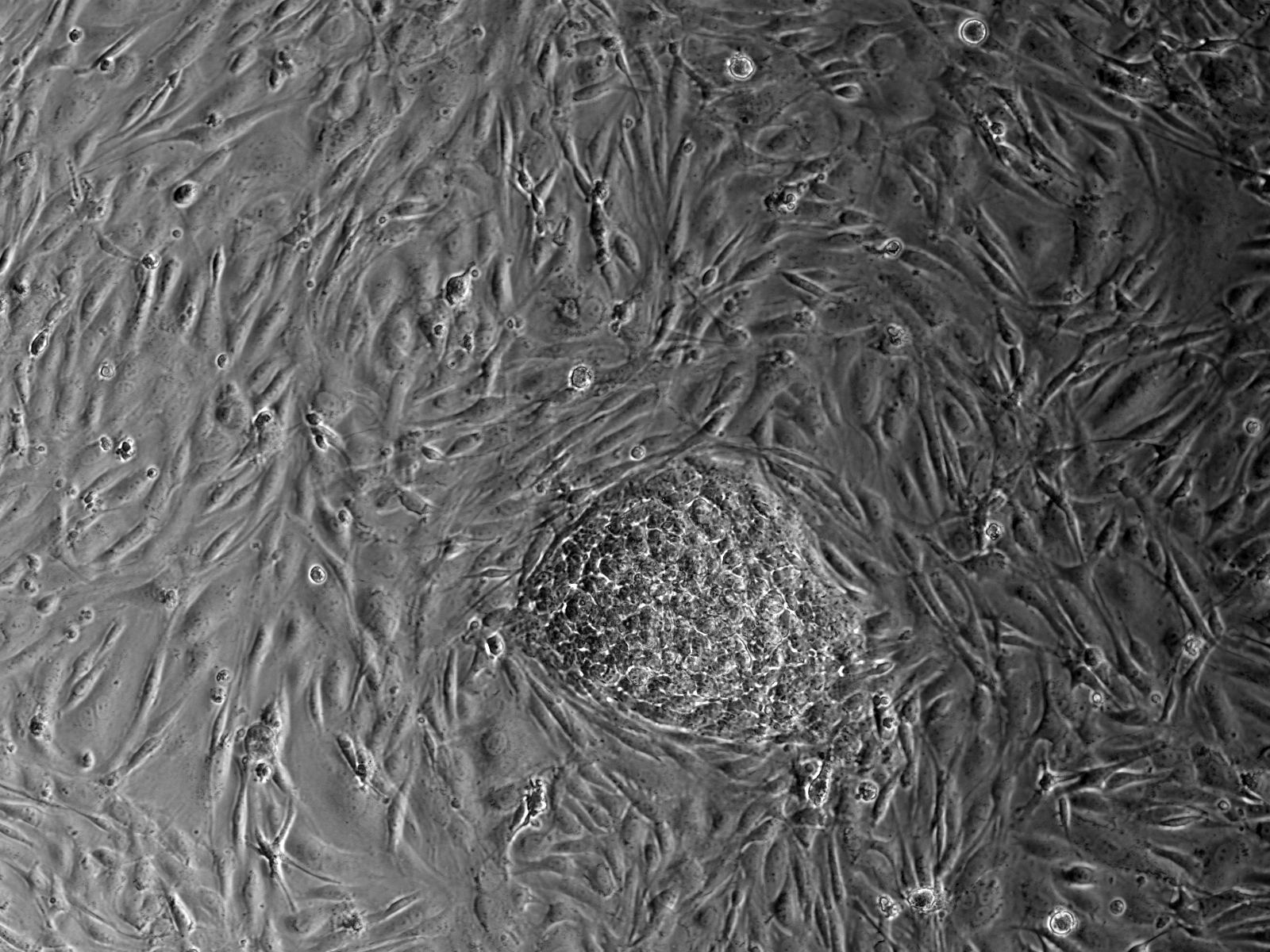

Supplement: Supplementary file 6 — Source data Fig. 2 [file 44318_2025_527_MOESM6_ESM.zip › Figure 2/2D/Region 1 explant 24h.tif]

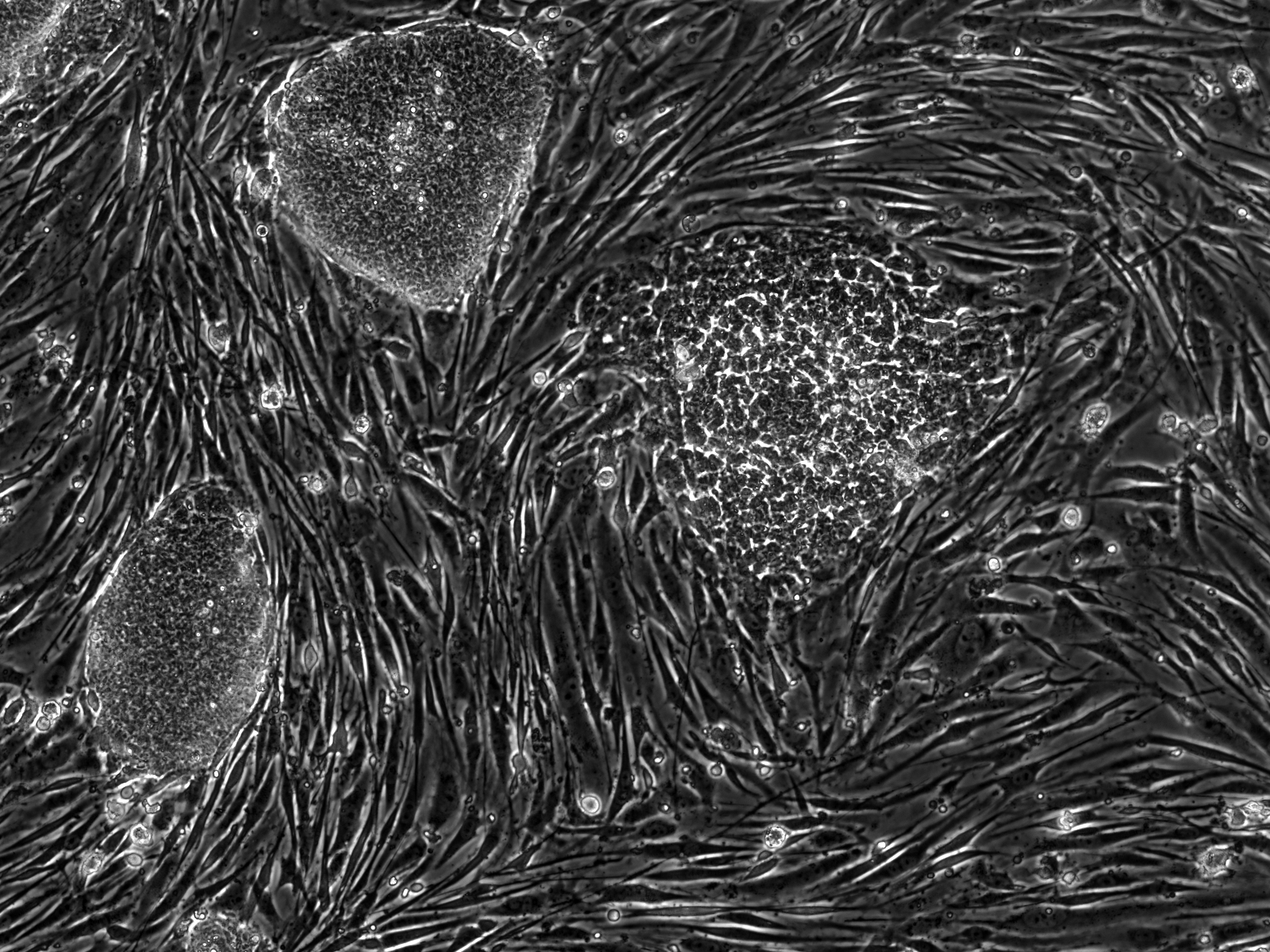

Supplement: Supplementary file 6 — Source data Fig. 2 [file 44318_2025_527_MOESM6_ESM.zip › Figure 2/2D/Region R explant 24h.tif]

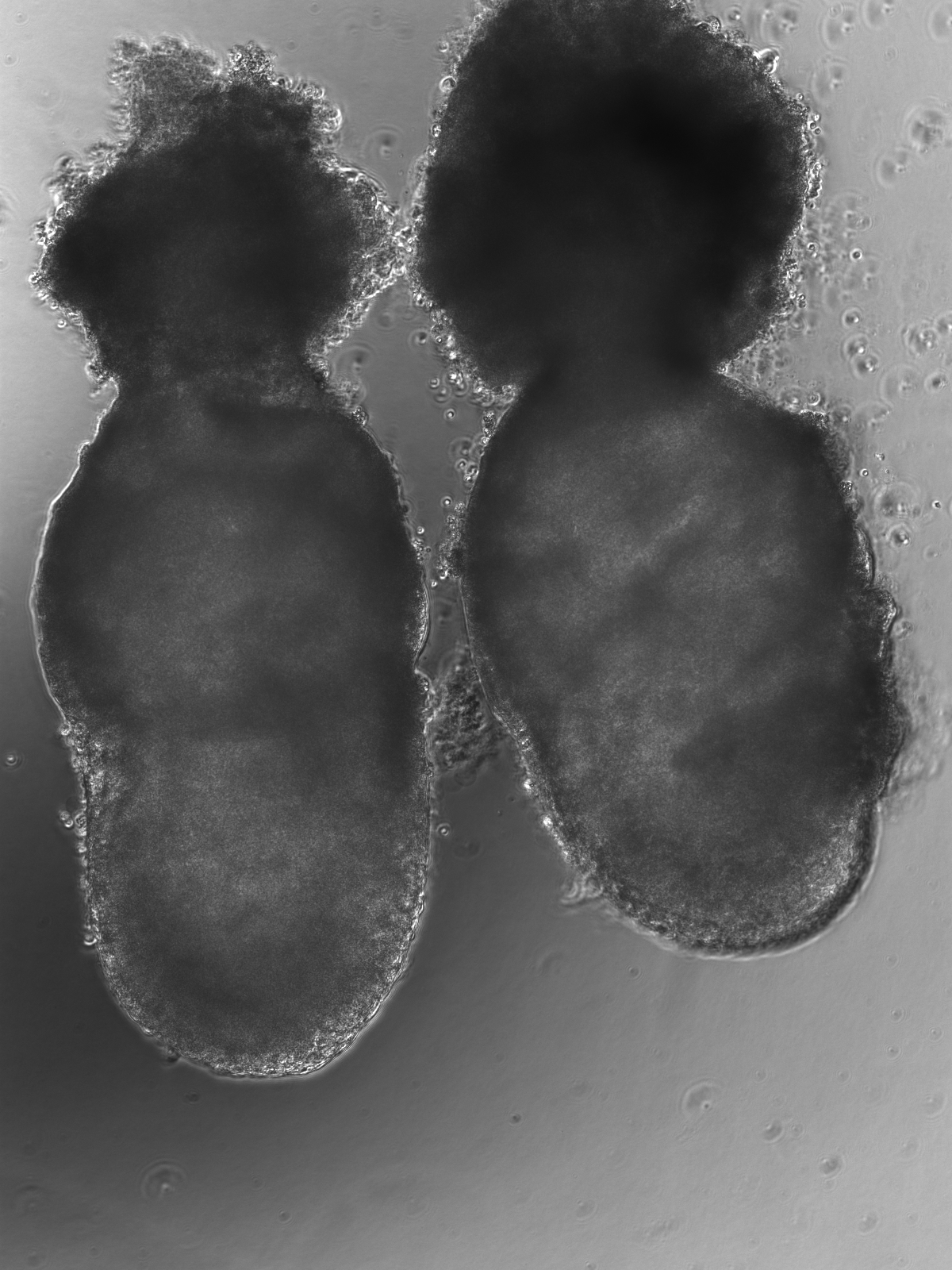

Supplement: Supplementary file 6 — Source data Fig. 2 [file 44318_2025_527_MOESM6_ESM.zip › Figure 2/2I/Embryo Sox2-GFP 2h30m.tif]

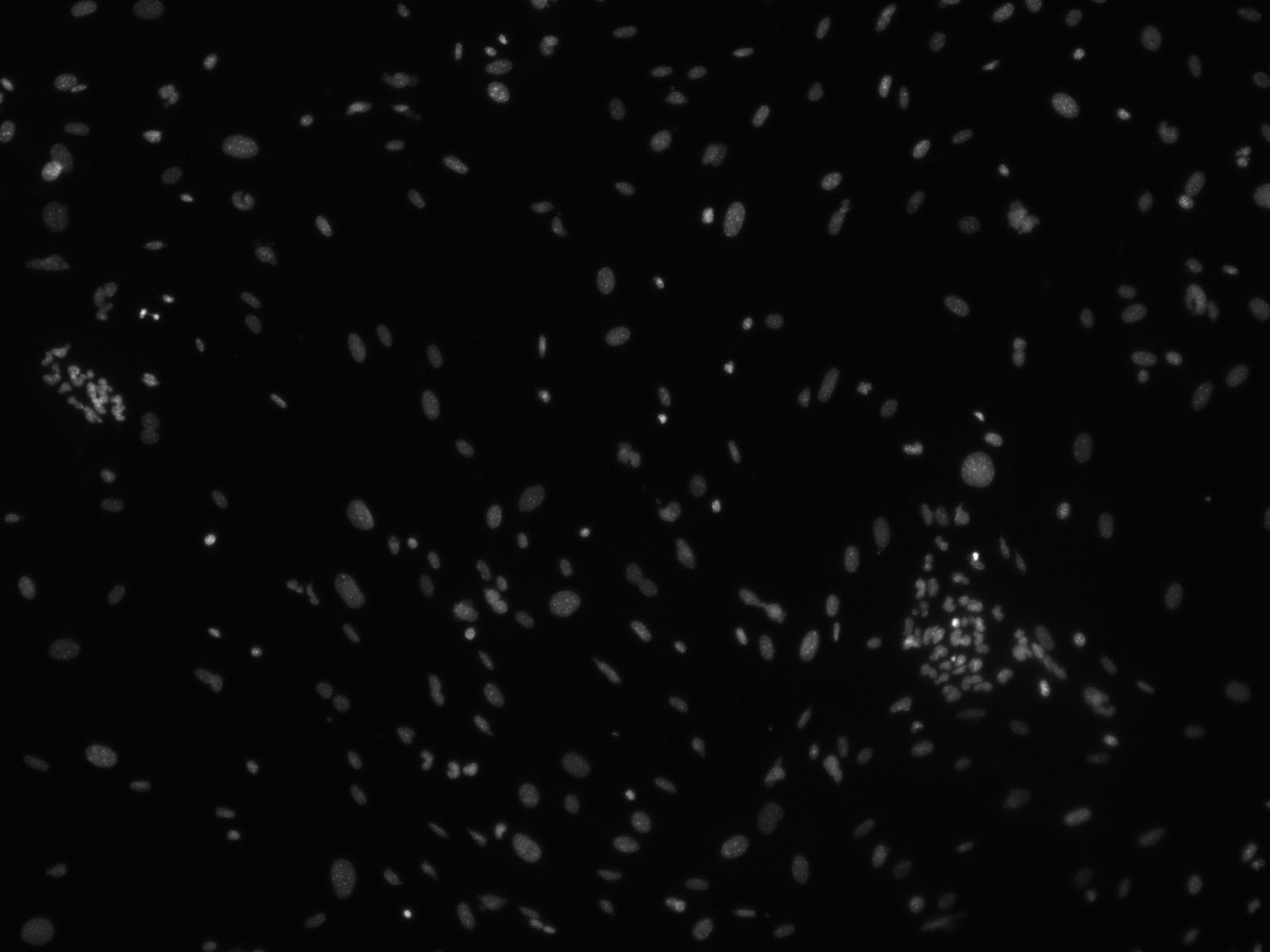

Supplement: Supplementary file 6 — Source data Fig. 2 [file 44318_2025_527_MOESM6_ESM.zip › Figure 2/2J/Region 1 GFP.tif]

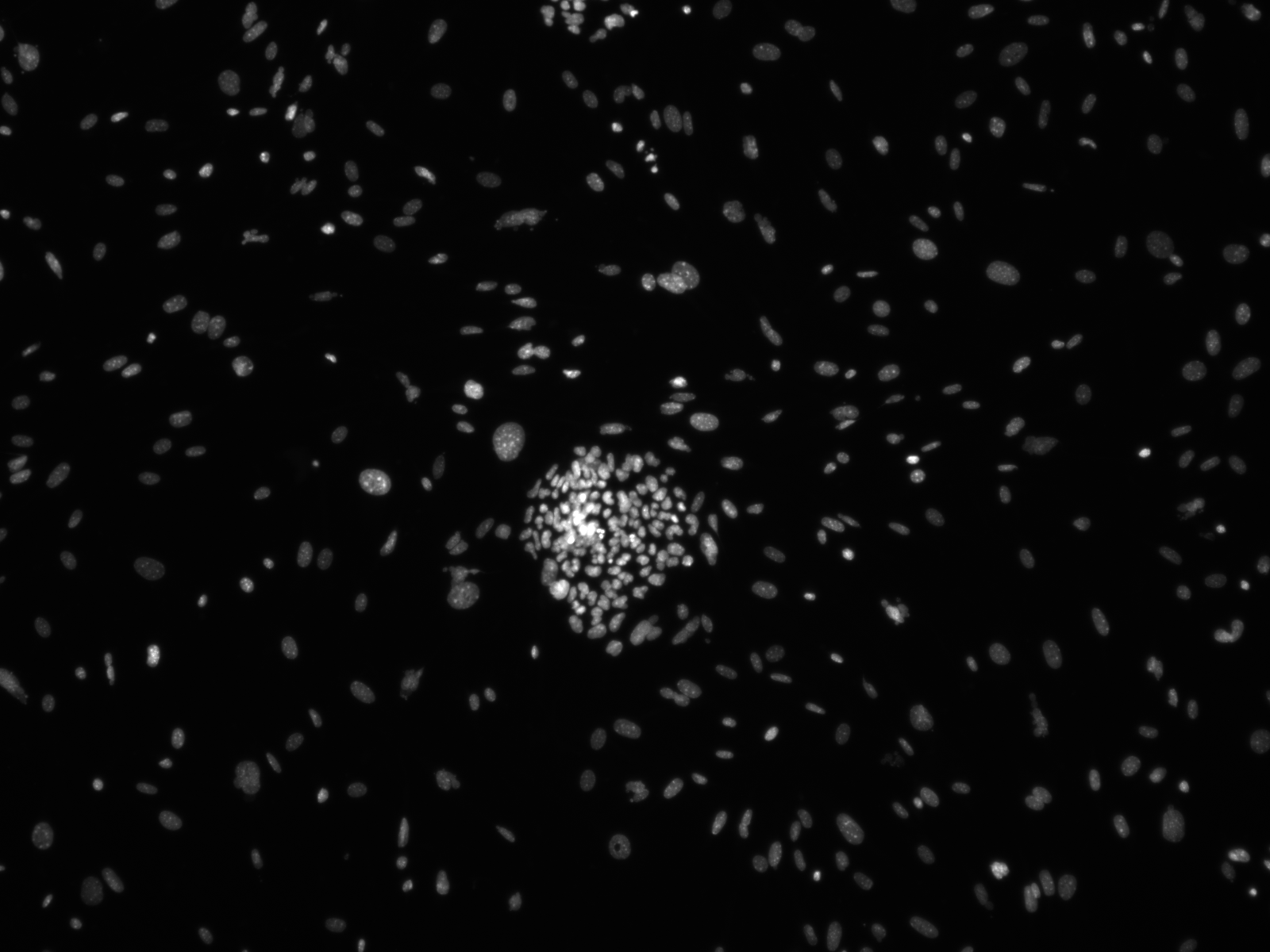

Supplement: Supplementary file 6 — Source data Fig. 2 [file 44318_2025_527_MOESM6_ESM.zip › Figure 2/2J/Region 1 Sox2-GFP.tif]

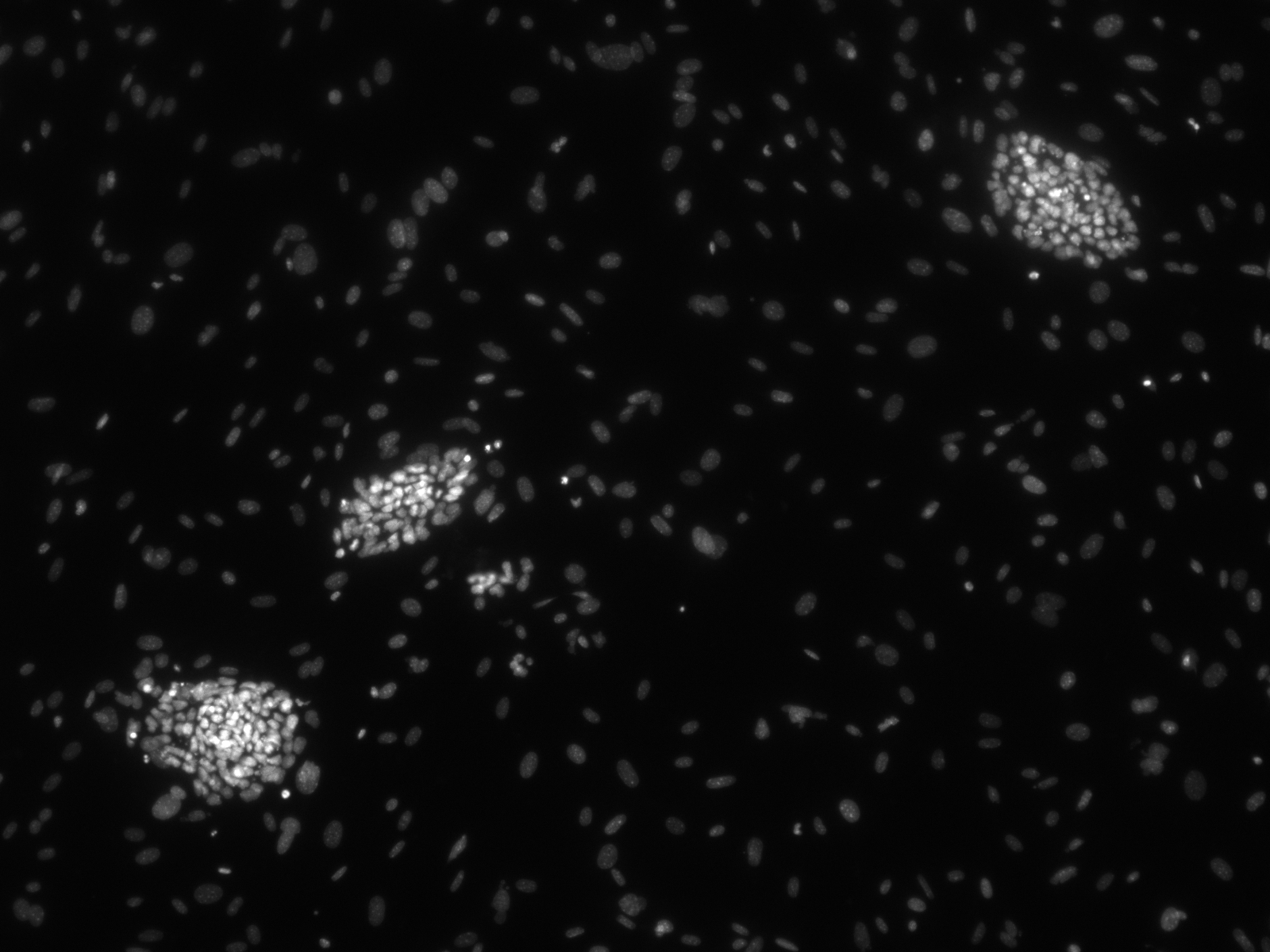

Supplement: Supplementary file 6 — Source data Fig. 2 [file 44318_2025_527_MOESM6_ESM.zip › Figure 2/2J/Region 8.tif]

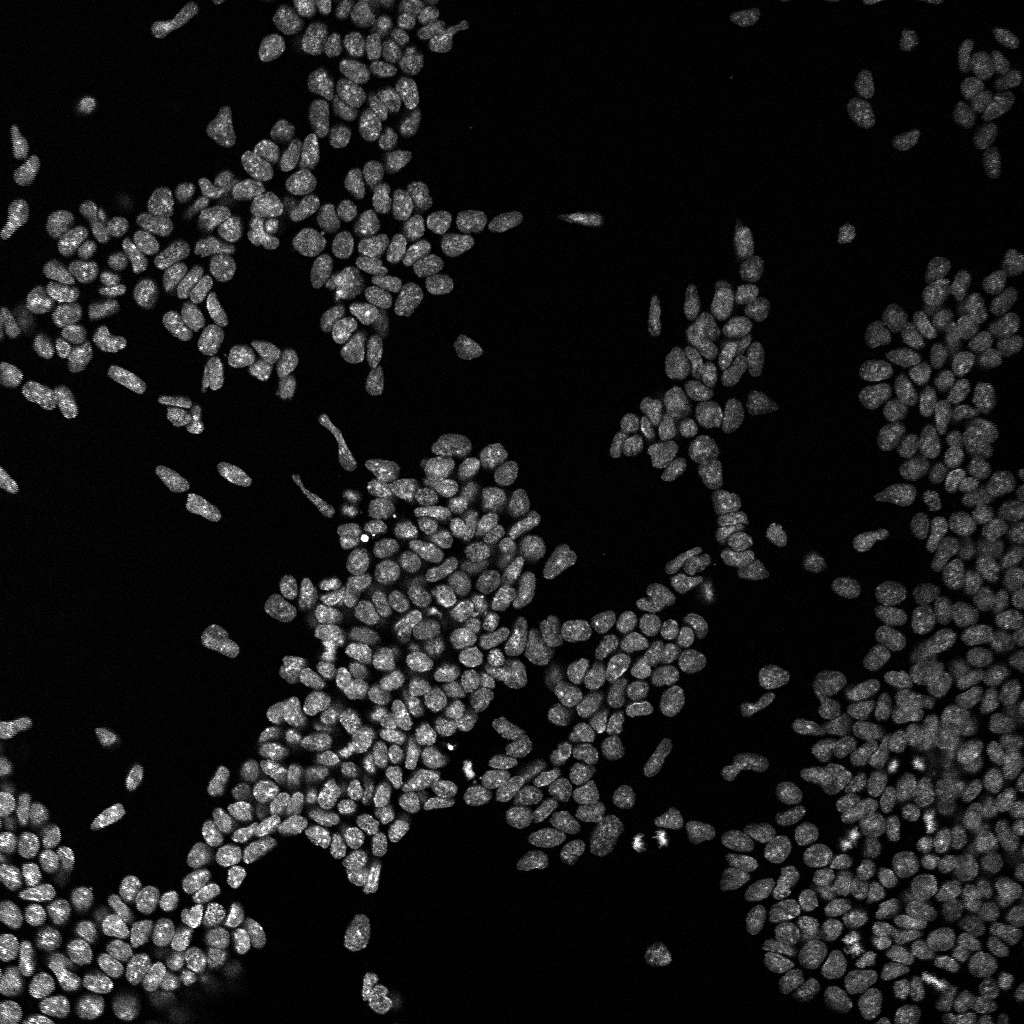

Supplement: Supplementary file 8 — Source data Fig. 4 [file 44318_2025_527_MOESM8_ESM.zip › Figure 4/4E/EpiLCs 0h.tif]

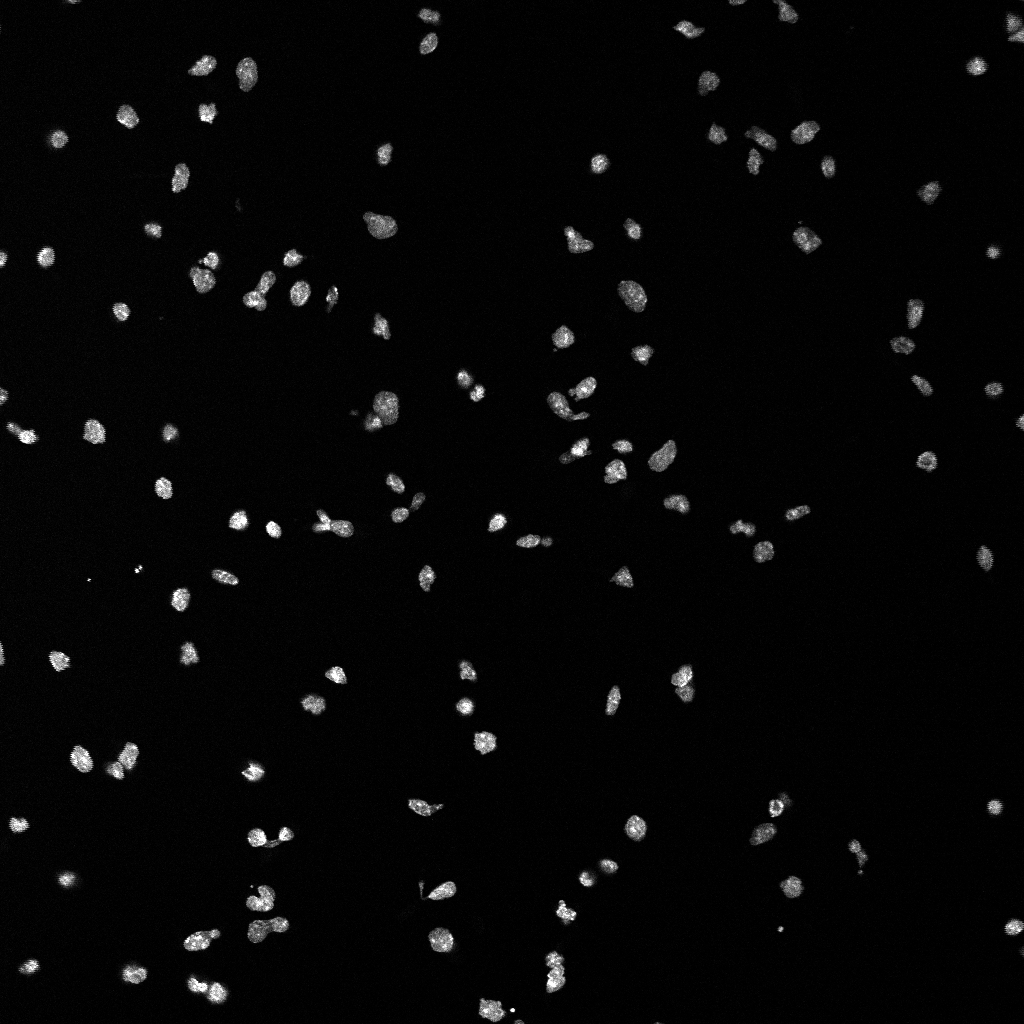

Supplement: Supplementary file 8 — Source data Fig. 4 [file 44318_2025_527_MOESM8_ESM.zip › Figure 4/4E/pEpiLC 1h.tif]

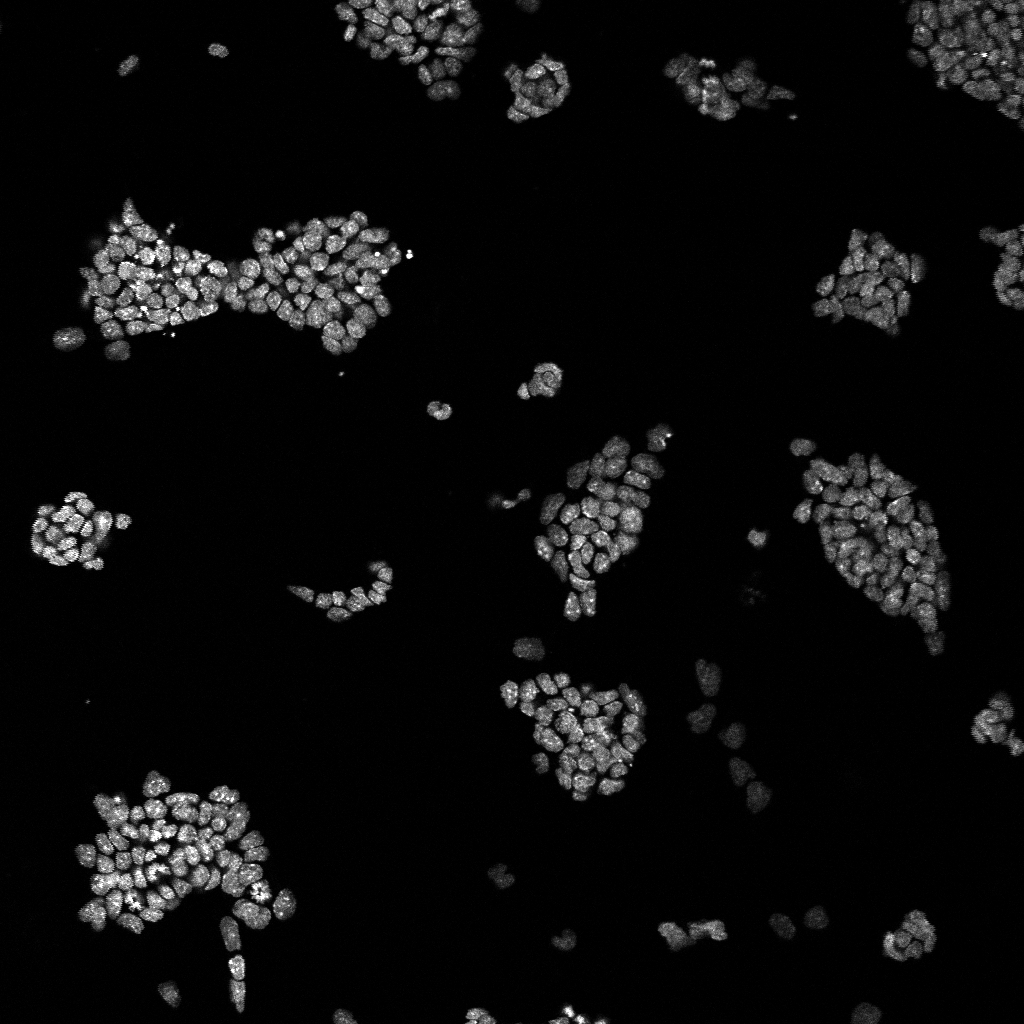

Supplement: Supplementary file 8 — Source data Fig. 4 [file 44318_2025_527_MOESM8_ESM.zip › Figure 4/4E/pEpiLC 24h.tif]

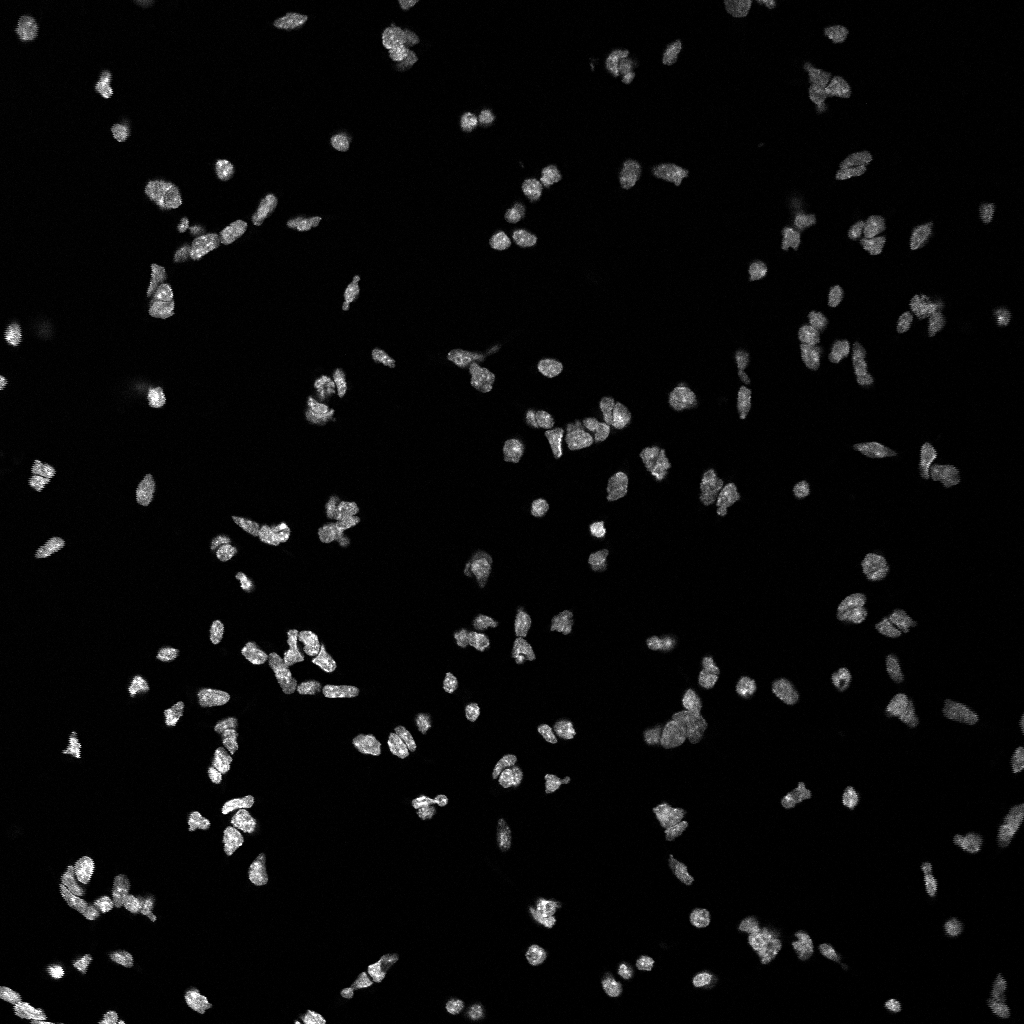

Supplement: Supplementary file 8 — Source data Fig. 4 [file 44318_2025_527_MOESM8_ESM.zip › Figure 4/4E/pEpiLC 3h.tif]

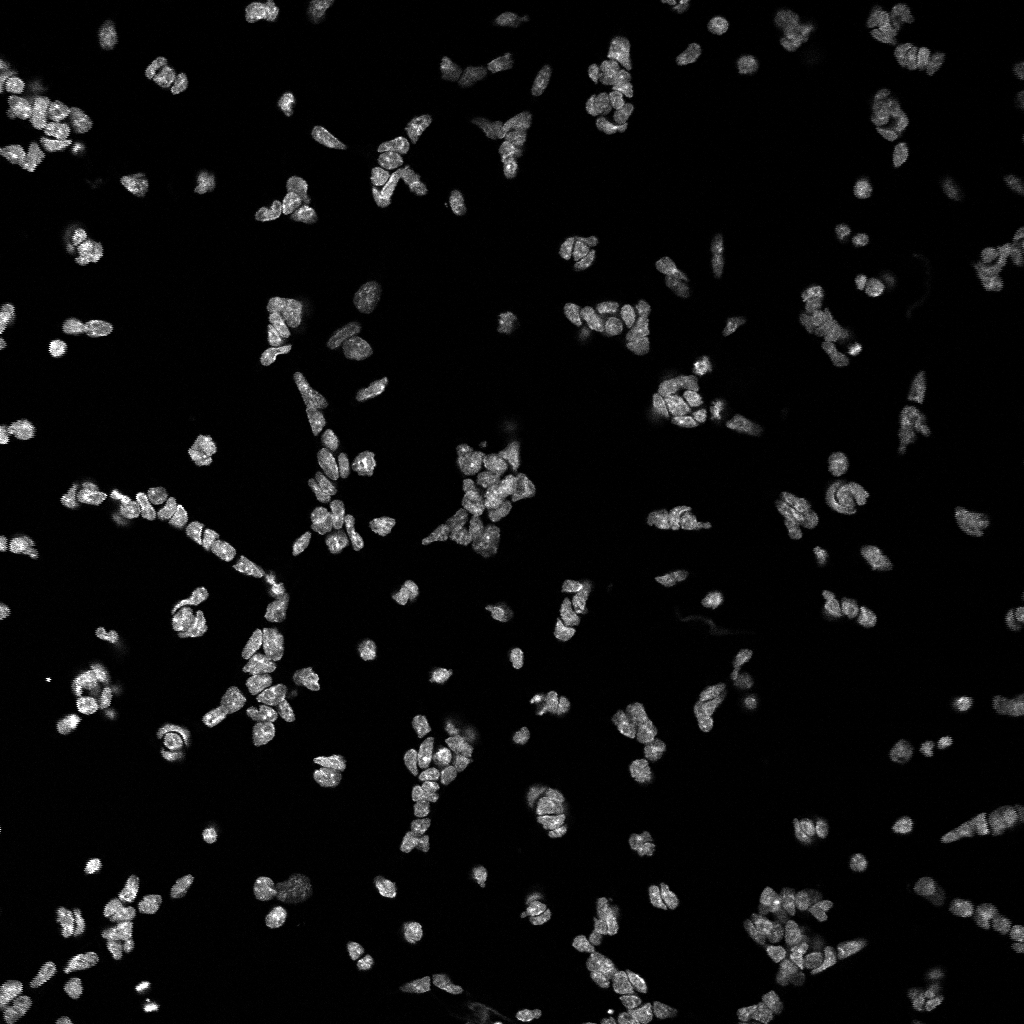

Supplement: Supplementary file 8 — Source data Fig. 4 [file 44318_2025_527_MOESM8_ESM.zip › Figure 4/4E/pEpiLC 6h.tif]

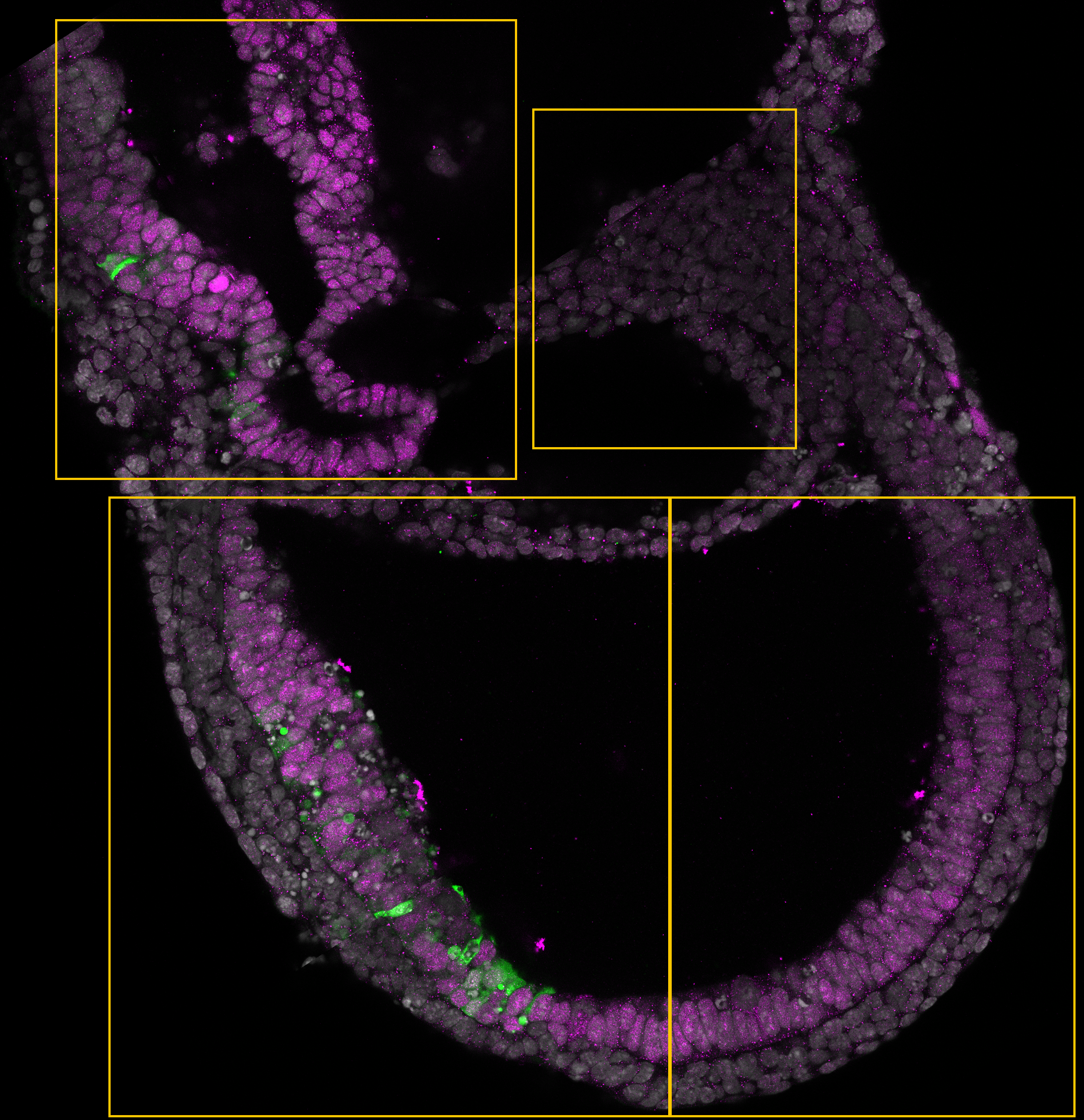

Supplement: Supplementary file 9 — Source data Fig. 5 [file 44318_2025_527_MOESM9_ESM.zip › Figure 5/5B/GFP2 regions.tif]

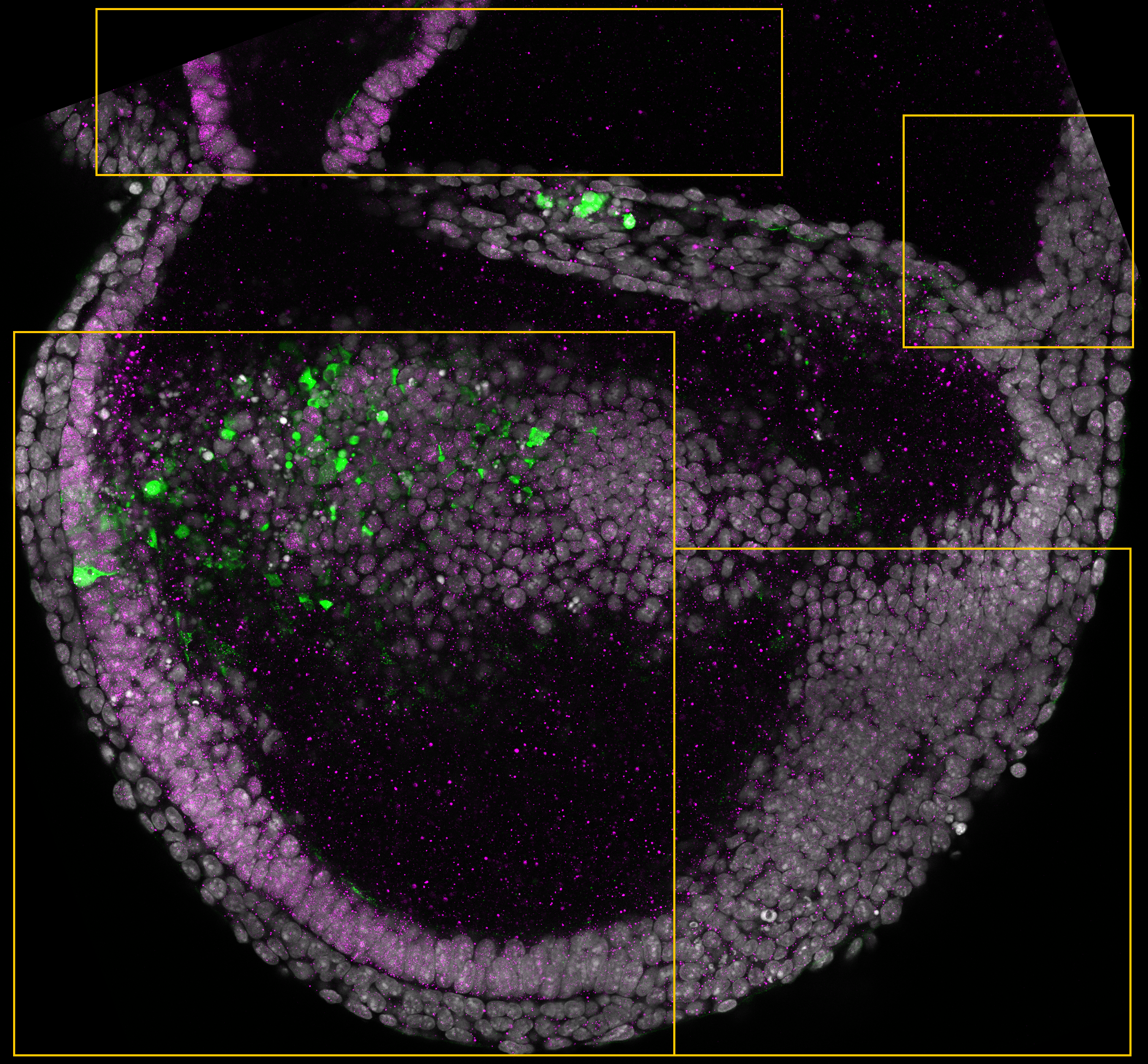

Supplement: Supplementary file 9 — Source data Fig. 5 [file 44318_2025_527_MOESM9_ESM.zip › Figure 5/5B/NANOG2 regions.tif]

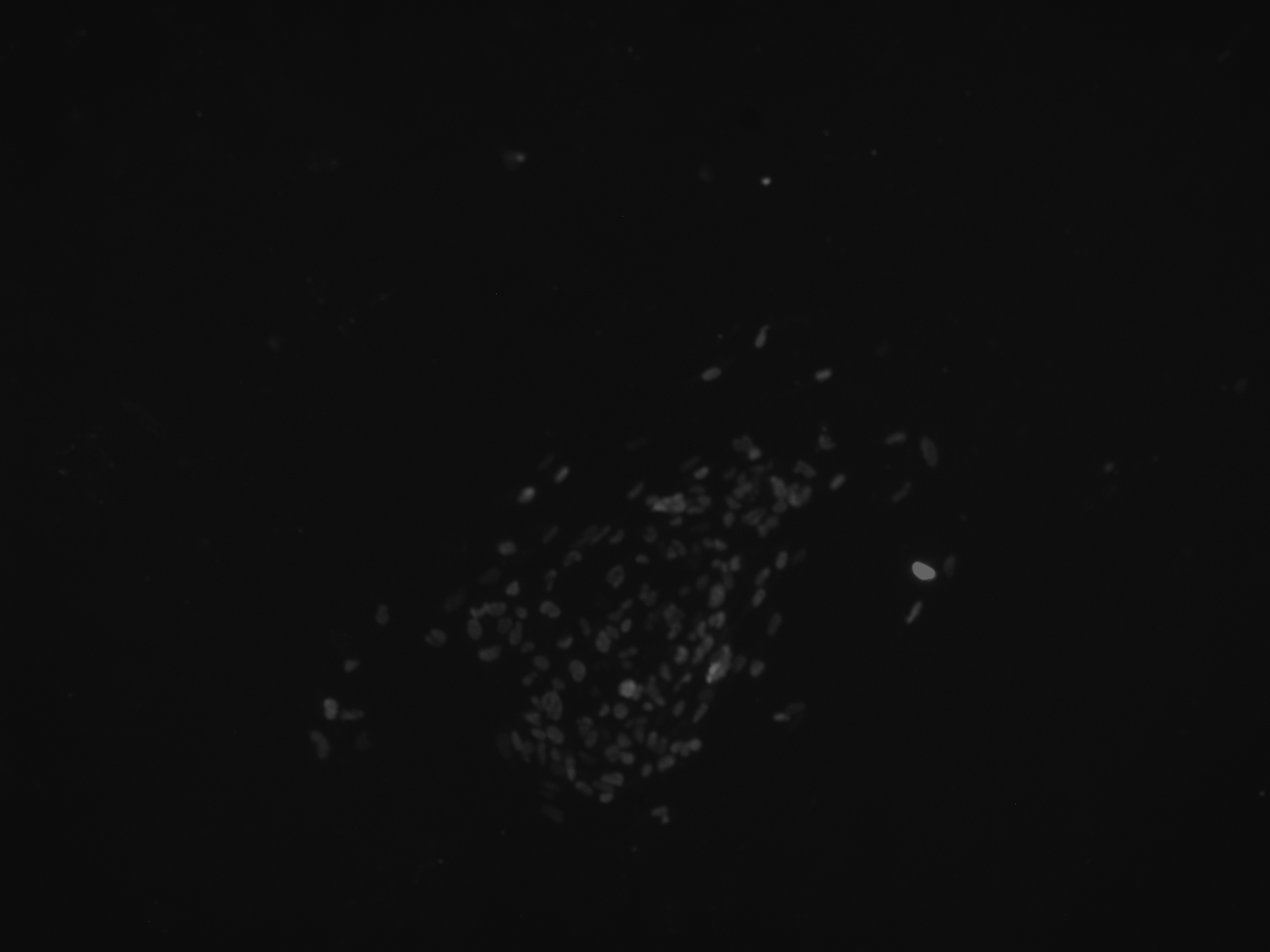

Supplement: Supplementary file 10 — Figure EV5F Source Data [file 44318_2025_527_MOESM10_ESM.zip › Forebrain Mesenchyme.tif]

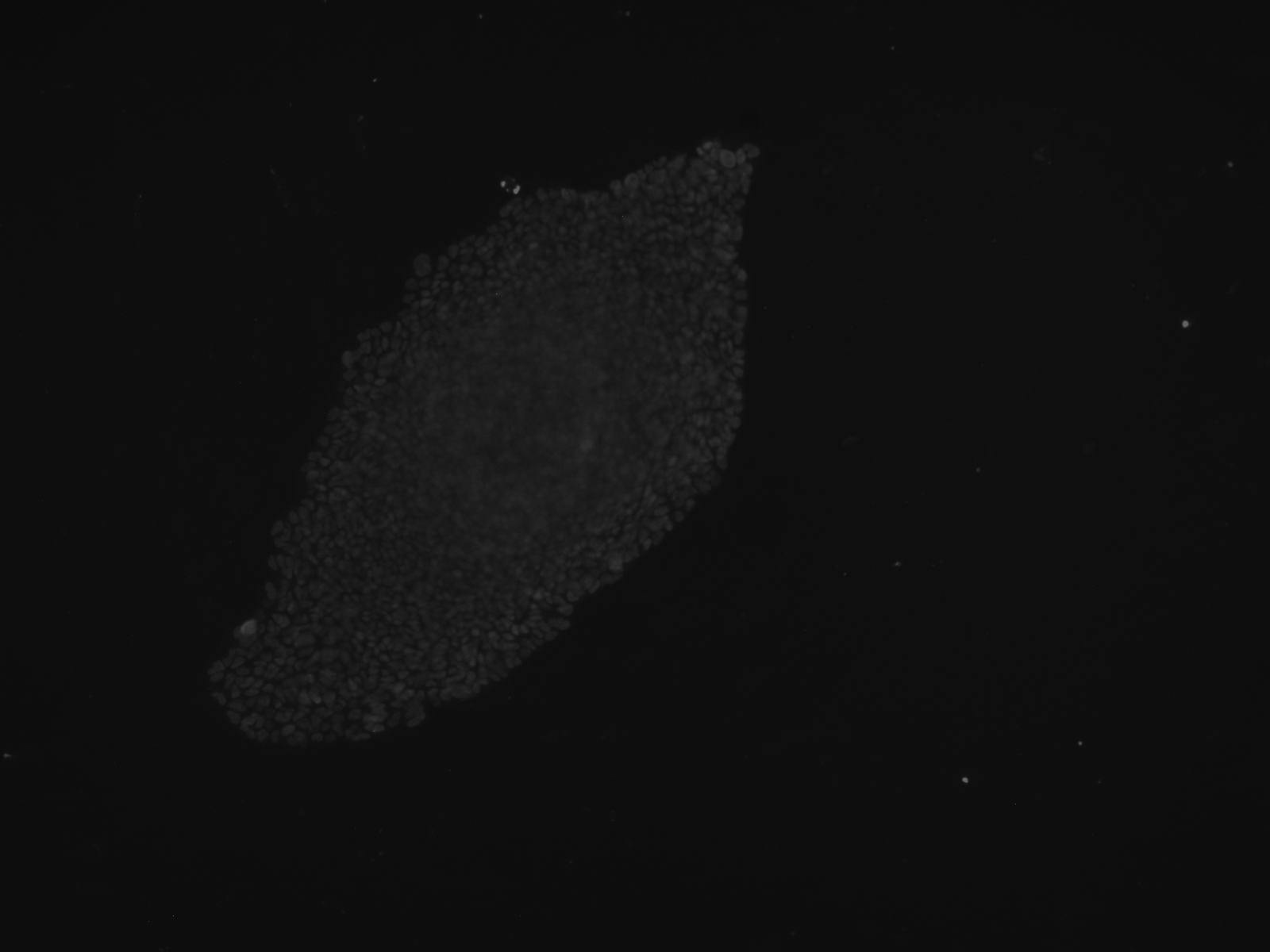

Supplement: Supplementary file 10 — Figure EV5F Source Data [file 44318_2025_527_MOESM10_ESM.zip › Primitive Streak Epithelium.tif]
