# Supplementary material for: NANOG is repurposed after implantation to repress Sox2 and begin pluripotency extinction
Source: EMBO J. 2025 Aug 18;44(19):5337–74. doi: 10.1038/s44318-025-00527-9 (PMC12488938; doi:10.1038/s44318-025-00527-9)
Supplement: Supplementary file 11 — Expanded View Figures [file 44318_2025_527_MOESM11_ESM.pdf]

Expanded View Figures

Figure EV1. Supporting Fig. 1.

(**A**) Whole-mount embryo immunofluorescence showing OCT4 (cyan), SOX2 (red), NANOG (green) and DAPI (grey). Embryos were imaged by confocal microscopy and are shown as maximum Z-stack projections. Scale bars represent 20 µm. (**B**) Quantitation of SOX2/OCT4 and SOX2/NANOG immunofluorescence; number of embryos (brackets) and cells analysed (top right of righthand plot) are indicated. Gates were set based on the background fluorescence levels in the negative regions (see Fig. EV1D for example) per embryo. Quantified ANIs are represented in a 5% contour plot showing all events. (**C**) Pearson's correlation coefficients (PMCC, r^2) were calculated between SOX2 and NANOG ANI distributions at E4.5 ($n = 8$) and E6.5 ($n = 7$). (**D**) Regions cropped for whole-mount immunofluorescence quantification of E3.5–5.5 and E7.5-7.75. A separate internal negative region (0') was used for OCT4 ANI quantification at E3.5. Whole embryo images were quantified for E3.5 to E5.5 embryos, embryonic regions E were quantified for E6.5 to E7.75 embryos. A superficial z-slice was included to show lateral cells in E7.5 embryos (inset).

© The Author(s)

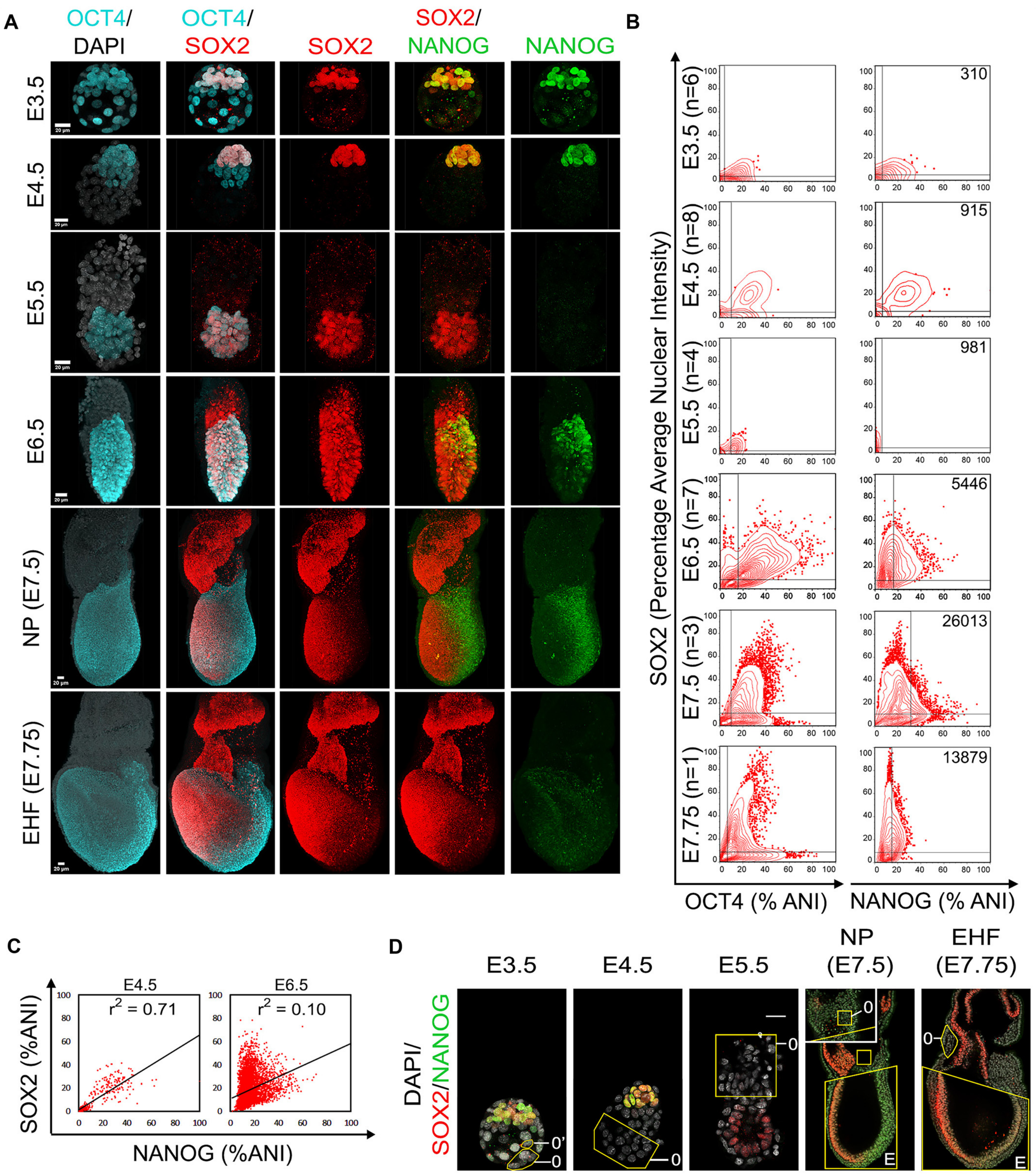
A
OCT4/ DAPI
OCT4/ SOX2
SOX2
SOX2/ NANOG
NANOG
E3.5
E4.5
E5.5
E6.5
NP (E7.5)
EHF (E7.75)
B
SOX2 (Percentage Average Nuclear Intensity)
E3.5 (n=6)
E4.5 (n=8)
E5.5 (n=4)
E6.5 (n=7)
E7.5 (n=3)
E7.75 (n=1)
310
915
981
5446
26013
13879
OCT4 (% ANI)
NANOG (% ANI)
C
E4.5
r2 = 0.71
E6.5
r2 = 0.10
SOX2 (%ANI)
NANOG (%ANI)
D
E3.5
E4.5
E5.5
NP (E7.5)
EHF (E7.75)
DAPI/ SOX2/NANOG
E

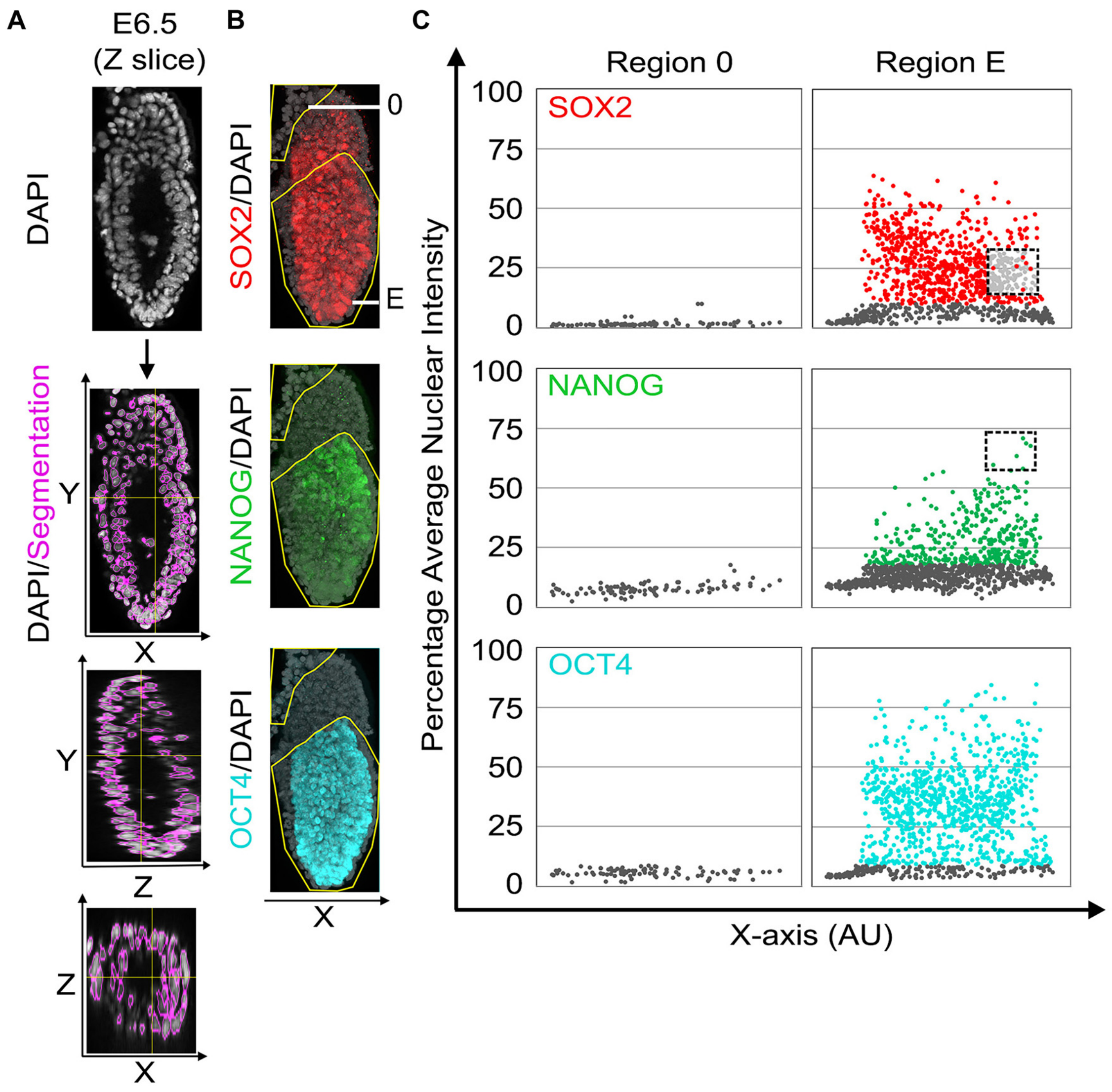

Figure EV2. Supporting Fig. 1.

(**A**) Workflow to quantify immunofluorescence per nucleus. The outline of each segment is shown (magenta). Orthogonal sections from a confocal Z-stack showing nucleus segmentation in three dimensions. (**B**) Regions cropped for whole-mount immunofluorescence quantification are shown by yellow outlines. An internal negative control region marked 0 was used to set gates for each embryo. (**C**) Average immunofluorescence signals from the three different channels shown in (**B**) were quantified by normalising to the nuclear volume to determine the average nuclear intensity (ANI) for each nucleus. ANIs were normalised to the maximal ANI per channel observed in all the embryos in the dataset shown in Fig. EV1A,B. ANIs from regions 0 and E are plotted along the x axis; the horizontal width of the corresponding images in (**B**). Nuclei in region E with ANI less than or equal to the maximum level observed in region 0 were considered negative (dark grey dots). Cells with the highest NANOG ANI (dashed black box in Nanog plot) in the proximal territory were assessed for SOX2, and the corresponding Sox2 ANI of these cells are shown (red inside dashed black box in SOX2 plot).

 © The Author(s)

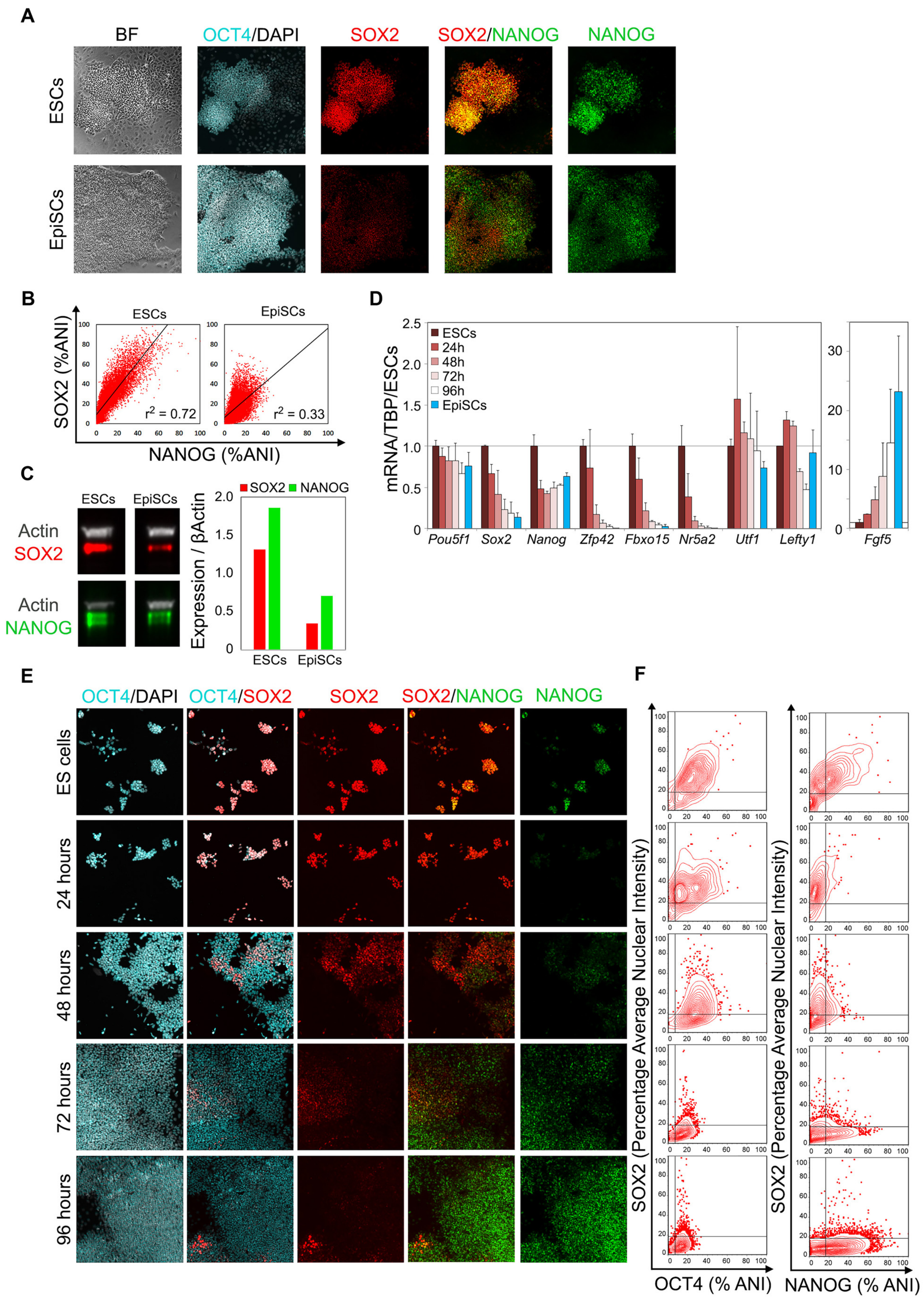
A
BF
OCT4/DAPI
SOX2
SOX2/NANOG
NANOG
ESCs
EpiSCs
B
ESCs
EpiSCs
SOX2 (%ANI)
NANOG (%ANI)
r2 = 0.72
r2 = 0.33
C
ESCs
EpiSCs
Actin
SOX2
Actin
NANOG
SOX2 NANOG
Expression / βActin
D
mRNA/TBP/ESCs
ESCs
24h
48h
72h
96h
EpiSCs
Pou5f1
Sox2
Nanog
Zfp42
Fbxo15
Nr5a2
Utf1
Lefty1
Fgf5
E
OCT4/DAPI
OCT4/SOX2
SOX2
SOX2/NANOG
NANOG
ES cells
24 hours
48 hours
72 hours
96 hours
F
SOX2 (Percentage Average Nuclear Intensity)
OCT4 (% ANI)
NANOG (% ANI)

Figure EV3. NANOG and SOX2 are partially segregated in EpiSCs.

(**A**) Immunostaining for OCT4 (cyan), SOX2 (red) and NANOG (green) in E14TG2a ESCs and in EpiSCs. Nuclei were stained with DAPI (grey) (representative of $n = 26$ fields in 2 experimental replicates). (**B**) PMCC, (r^2) values were calculated between SOX2 and NANOG ANI distributions in ESCs ($n = 13011$ cells) and EpiSCs ($n = 9793$ cells analysed). (**C**) Total protein was extracted from ESCs and EpiSCs and analysed by immunoblotting for SOX2 (red), NANOG (green) and β-ACTIN (grey), lanes were cropped from the same blot. Right: quantification of SOX2 or NANOG band intensity relative to that of β-ACTIN. Shown is a representative of two independent replicate experiments. (**D**) Quantitative RT-PCR was performed for *Pou5f1*, *Sox2*, *Nanog*, *Zfp42 (Rex1)*, *Fbxo15*, *Nr5a2*, *Utf1*, *Lefty1* and *Fgf5* transcripts at the indicated times during the ESC to EpiSC transition. Transcript levels were normalised to TBP and plotted relative to ESC levels (black horizontal line). Error bars denote standard deviation (ESC, $n = 3$, 24-96 h, $n = 2$, EpiSC, $n = 4$ experimental replicates). (**E**) Immunostaining for OCT4 (cyan), SOX2 (red) and NANOG (green) during the ESC to EpiSC transition in E14TG2a cells. Nuclei were stained with DAPI (grey). (**F**) ANIs of SOX2/OCT4 and SOX2/NANOG were quantified for each 24 h time-point and represented in a 5% contour plot showing all events.

 © The Author(s)

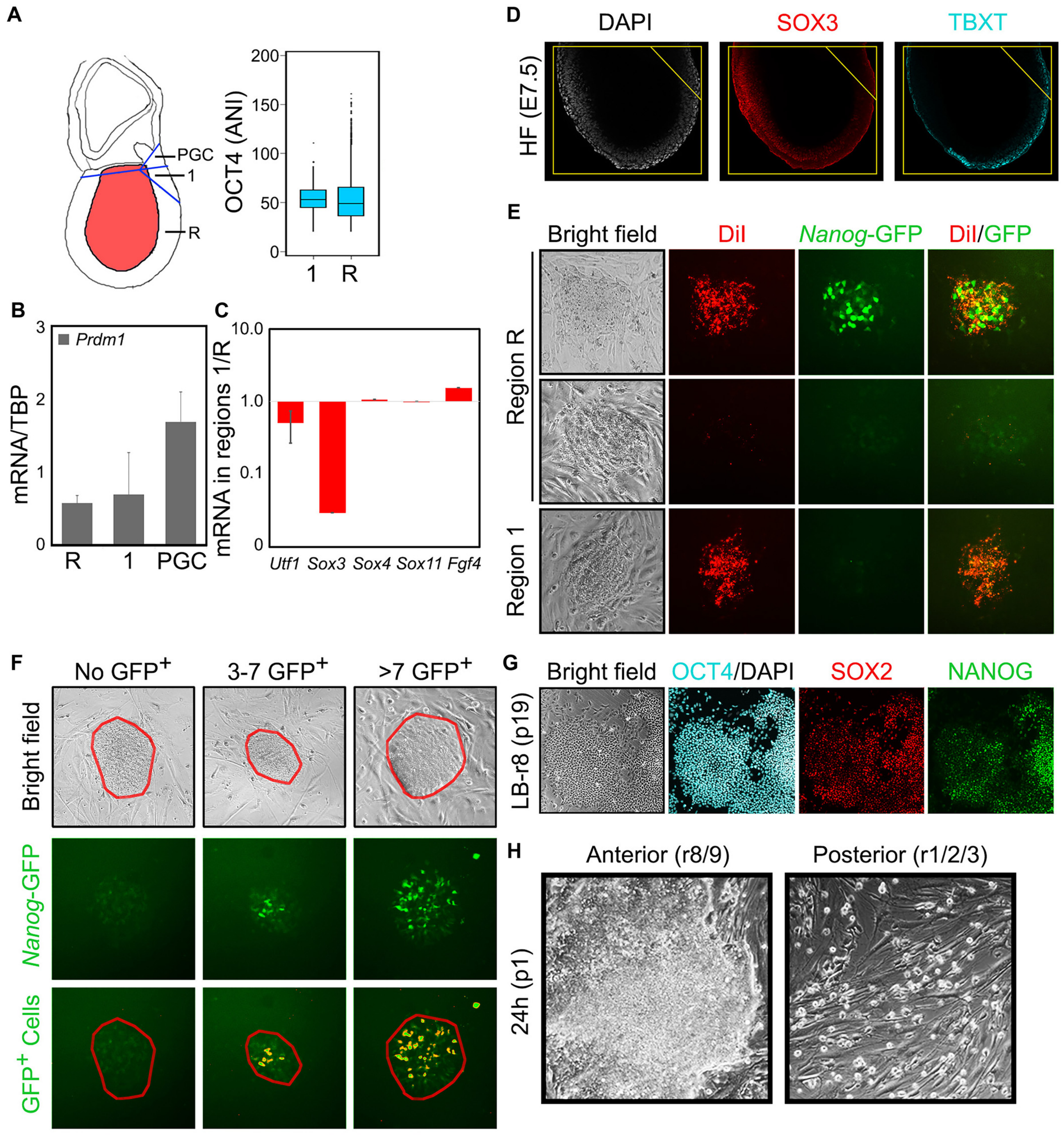

I

Region	Construct	N(exp)	N(fields)	N(clusters)	N(GFP+)	N(SOX2+)	N(NANOG+)
1	GFP	3	8	16	11	2	1
1	Sox2::GFP	3	10	26	13	16	18
8	GFP	3	7	18	0	11	11
8	Sox2::GFP	3	6	12	2	5	7

Figure EV4. Supporting Fig. 2.

(**A**) Left: Schematic diagram showing regions 1, R and 'PGC' for dissection. Red shading denotes DiI label injected into the amniotic cavity. Right: ANI distribution of OCT4 immunofluorescence in regions 1 and R of two representative embryos. Background ANI values determined using the internal negative control region 0 (Fig. EV1D) for each embryo were excluded in this plot. (**B**) QRT-PCR analysis of *Prdm1* (*Blimp1*) mRNA levels in regions 1, R and a PGC-containing region proximal to region 1 that includes the base of the allantois ($n = 2$). Error bars = SD. (**C**) Quantitative RT-PCR analysis of *Utf1, Sox3, Sox4*, *Sox11*, and *Fgf4* mRNA levels in regions 1 and R from dissected E7.5 embryos. For *Utf1*, $n = 2$; others, $n = 1$. Error bars = SD. (**D**) SOX3 (red) and TBXT (cyan) immunofluorescence at EHF stage. (**E**) *Nanog*-GFP was only observed in cells labelled with DiI after 24 h in culture (top row versus middle). Triturated region R tissue was diluted 1:10 prior to explantation; Region 1 was undiluted. (**F**) Colonies identified morphologically in bright field (red outline; top row) were examined for *Nanog*-GFP fluorescence (middle row). Using a constant threshold of green intensity, areas containing pixels with green intensity above this threshold were outlined (bottom row). The number of outlined areas in each outlined colony were scored and grouped into categories with no GFP$+$, 3–7 GFP^{+} or more than 7 GFP^{+} cells. Colonies with fewer than 3 GFP$+$ cells were indistinguishable from those containing only debris and were excluded from analysis. (**G**) Feeder-free EpiSCs (p19) were derived from expanding explants from one single region 8 of an LB-stage embryo (LBr8.3 cells). These cells were positive for OCT4 (cyan), SOX2 (red) and NANOG (green) immunofluorescence; (grey = DAPI). (**H**) The morphologies of representative colonies from the indicated regions 24 h after the first passage. (**I**) Quantification of explanted colonies (clusters) derived from indicated embryo regions expressing GFP, SOX2 and/or NANOG in ($n = 3$) electroporation experiments as depicted in Fig 2J. Numbers of total fields of view per condition are indicated.

 © The Author(s)

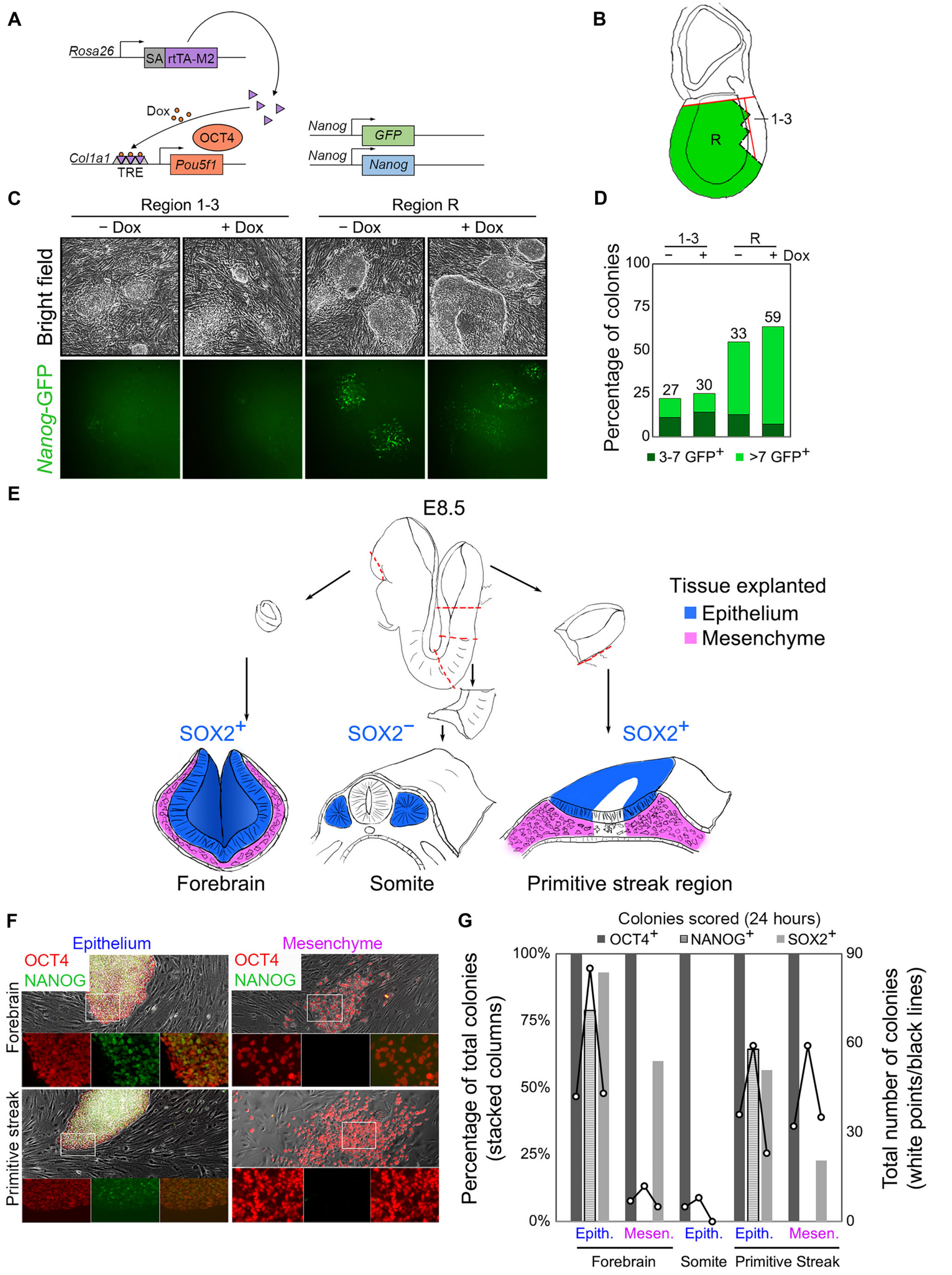
A
Rosa26
SA
rtTA-M2
Dox
OCT4
Col1a1
TRE
Pou5f1
Nanog
GFP
Nanog
Nanog
B
R
1-3
C
Region 1-3
Region R
− Dox
+ Dox
− Dox
+ Dox
Bright field
Nanog-GFP
D
Percentage of colonies
1-3
R
+ Dox
27
30
33
59
3-7 GFP+
>7 GFP+
E
E8.5
Tissue explanted
Epithelium
Mesenchyme
SOX2+
SOX2−
SOX2+
Forebrain
Somite
Primitive streak region
F
Epithelium
Mesenchyme
OCT4
NANOG
Forebrain
Primitive streak
G
Colonies scored (24 hours)
OCT4+
NANOG+
SOX2+
Percentage of total colonies (stacked columns)
Total number of colonies (white points/black lines)
Epith.
Mesen.
Epith.
Epith.
Mesen.
Forebrain
Somite
Primitive Streak

© The Author(s)

◀ **Figure EV5. Enforced OCT4 expression does not restore pluripotency to posterior epiblast.**

(**A**) The doxycycline (Dox)-inducible OCT4 system. Ectopic *Pou5f1*/OCT4 expression is under the control of a tetracycline response element (TRE) in a transgene targeted to the *Col1a1* locus. The reverse tetracycline transactivator (rtTA) is constitutively expressed from the *Rosa26* locus after a splice acceptor (SA). One endogenous allele of *Nanog* coding sequence is replaced with GFP open reading frame (ORF). (**B**) Posterior (regions 1–3) and remainder (R) epiblast were explanted. Green domain is the pluripotent domain identified in Fig. 2F. (**C**) Representative GFP fluorescence images of explants at 24 h +/− Dox from regions 1–3 or region R. (**D**) Colony quantitation based on GFP expression level (Fig. EV4F). Total colony numbers analysed in 3 experimental replicates are indicated above each bar. (**E**) Forebrain, somites and primitive streak regions were dissected from 4 independent E8.5 somite-stage embryos (red dashed lines). The epithelium (blue) and mesenchyme (magenta) layers were separated in forebrain and primitive streak regions; in the somite region only the epithelial somites were explanted. (**F**) Immunofluorescence analysis of forebrain and primitive streak explants for OCT4 (red) and NANOG (green). Bright-field and immunofluorescence images are overlaid to show expression in explanted cells. (**G**) Colonies from each explant were scored for the presence of OCT4, NANOG or SOX2-expressing cells and are displayed as percentages of total colonies (stacked columns). The total colony number (sum of 2 experiments) is plotted on a secondary axis (black lines with white points). Source data are available online for this figure.

 © The Author(s)

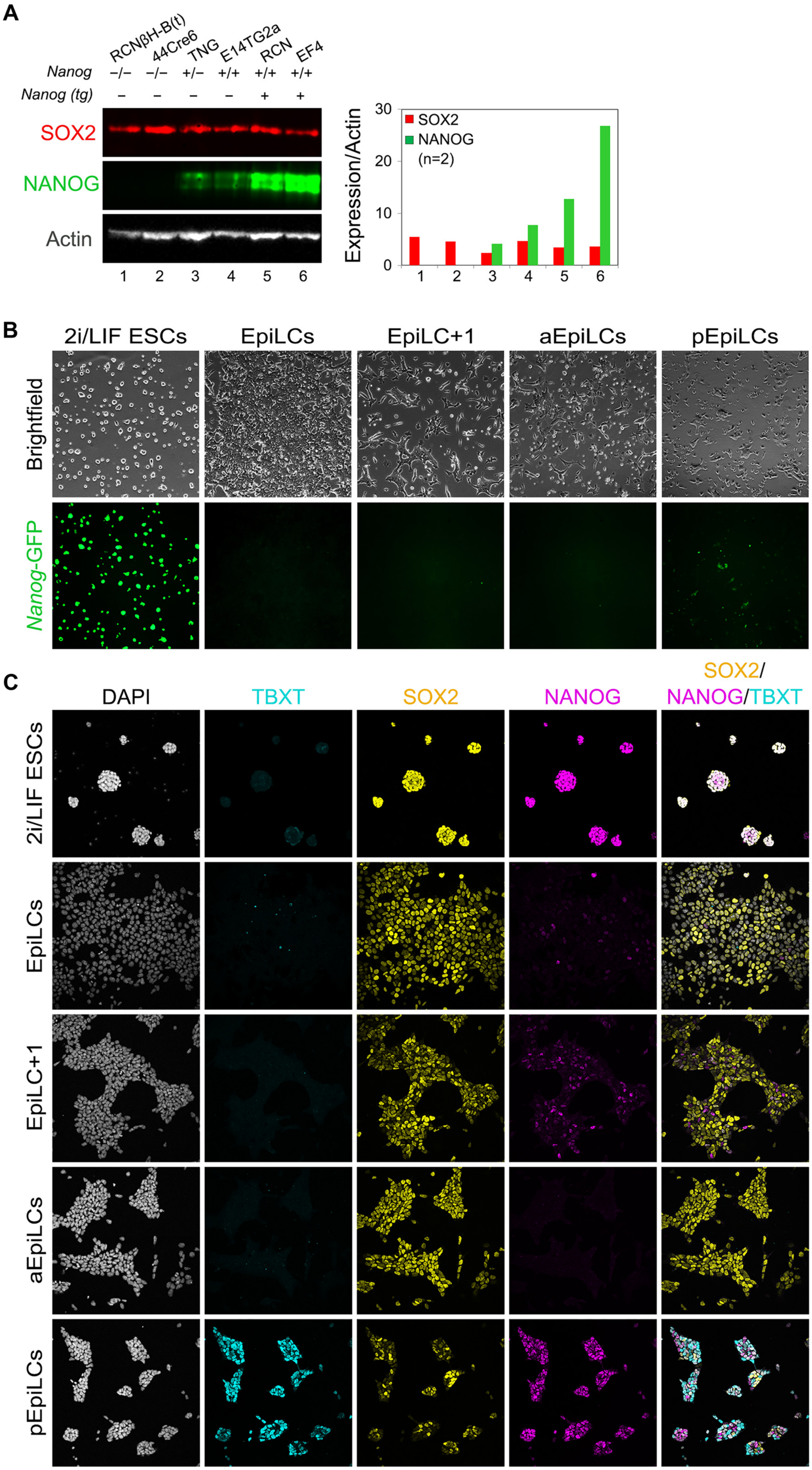
A
RCNβH-B(t)
44Cre6
TNG
E14TG2a
RCN
EF4
Nanog −/− −/− +/− +/+ +/+ +/+
Nanog (tg) − − − − + +
SOX2
NANOG
Actin
1 2 3 4 5 6
Expression/Actin
SOX2
NANOG
(n=2)
0
10
20
30
B
2i/LIF ESCs
EpiLCs
EpiLC+1
aEpiLCs
pEpiLCs
Brightfield
Nanog-GFP
C
DAPI
TBXT
SOX2
NANOG
SOX2/NANOG/TBXT
2i/LIF ESCs
EpiLCs
EpiLC+1
aEpiLCs
pEpiLCs

Figure EV6. Supporting Fig. 3.

(**A**) Left, immunoblot analysis of SOX2 (red), NANOG (green) and β-ACTIN (grey) in ESCs overexpressing NANOG (EF4 and RCN), *Nanog*$^{+/+}$ (E14TG2a), *Nanog*$^{+/-}$ (TNG) and *Nanog*$^{-/-}$ (44Cre6 and RCNβH-(t)) ESCs. Right, quantification of SOX2 (red) or NANOG (green) band intensity relative to that of β-ACTIN. Shown is a representative of two independent replicate experiments. (**B**) Morphology and GFP expression of *Nanog*-GFP cells in the indicated states (see Fig. 3A). (**C**) Immunofluorescence of NANOG (magenta), SOX2 (yellow) and TBXT (cyan) in E14Tg2a cells in the indicated states (see Fig. 3A).

 © The Author(s)

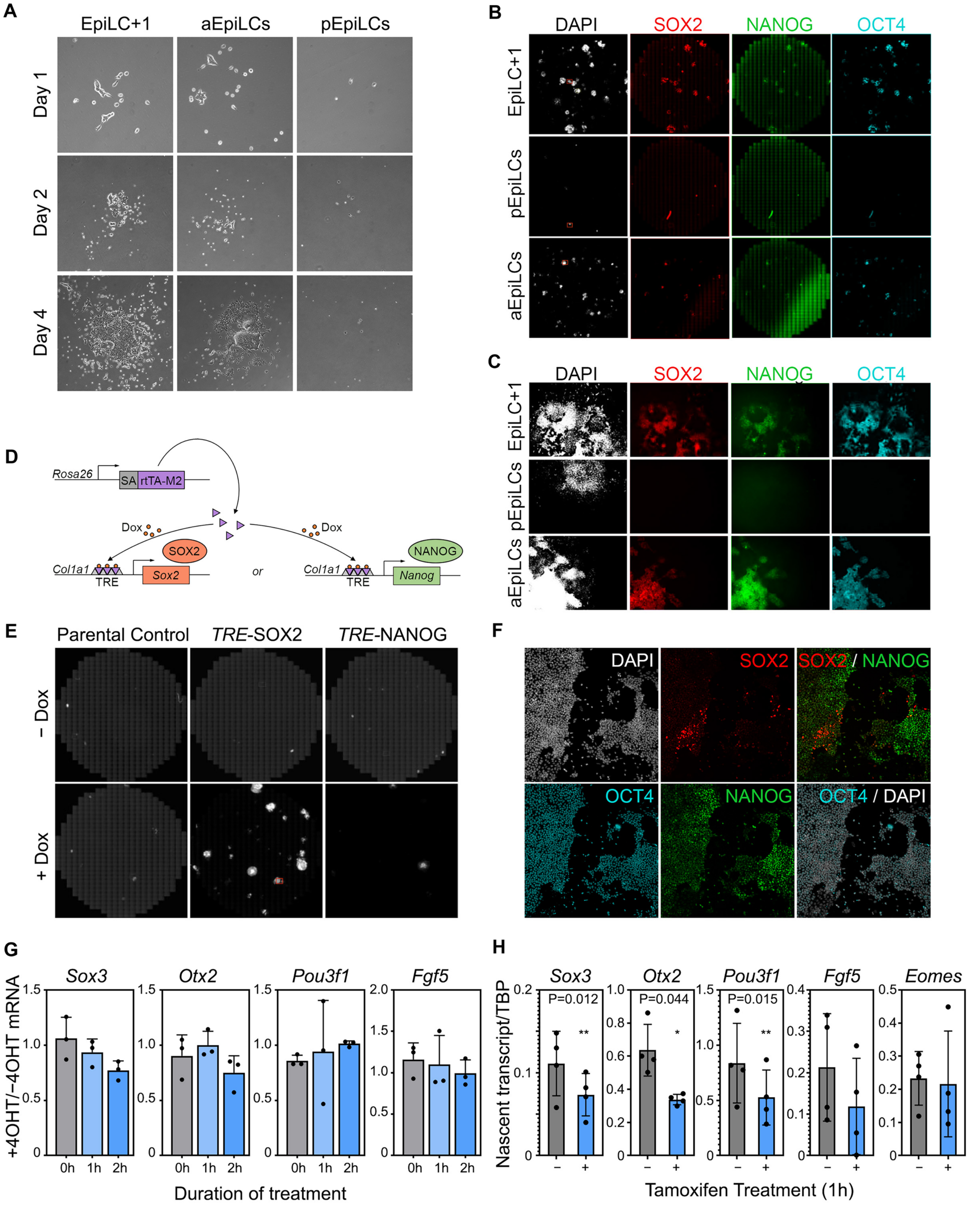
A
EpiLC+1
aEpiLCs
pEpiLCs
Day 1
Day 2
Day 4
B
DAPI
SOX2
NANOG
OCT4
EpiLC+1
pEpiLCs
aEpiLCs
C
DAPI
SOX2
NANOG
OCT4
EpiLC+1
pEpiLCs
aEpiLCs
D
Rosa26
SA
rtTA-M2
Dox
Dox
SOX2
NANOG
Col1a1
TRE
Sox2
or
Col1a1
TRE
Nanog
E
Parental Control
TRE-SOX2
TRE-NANOG
− Dox
+ Dox
F
DAPI
SOX2
SOX2 / NANOG
OCT4
NANOG
OCT4 / DAPI
G
+4OHT/−4OHT mRNA
Sox3
Otx2
Pou3f1
Fgf5
0h 1h 2h
Duration of treatment
H
Nascent transcript/TBP
Sox3
Otx2
Pou3f1
Fgf5
Eomes
P=0.012
P=0.044
P=0.015
Tamoxifen Treatment (1h)

© The Author(s)

Figure EV7. Supporting Fig. 3.

(**A**) Typical colony morphologies during the EpiSCs colony-forming assay seeded from the three indicated starter populations (see Fig. 3A). (**B**) Immunofluorescence of SOX2, OCT4 and NANOG at day 7 of the EpiSC colony-forming assay showing a representative plate-scanned area of 9.62 cm^2. (**C**) High magnification view of the fields indicated by red squares in (**B**), showing SOX2 (red), OCT4 (cyan) and NANOG (green) fluorescence channels. (**D**) The doxycycline (Dox)-inducible SOX2 or NANOG system. Ectopic SOX2 or NANOG expression is achieved after recombination-mediated cassette exchange (RMCE) of a cargo vector was achieved in parental KH2 cells to deliver *Sox2* or *Nanog* sequences under the control of a tetracycline response element (TRE) in the *Col1a1* locus. The reverse tetracycline transactivator (rtTA) is constitutively expressed from the *Rosa26* locus after a splice acceptor (SA). (**E**). DAPI staining at day 8 of the EpiSCs colony-forming assay (see Fig. 3D) showing a representative area of 9.62 cm^2 for the indicated cell line without (−) or with (+) doxycycline. (**F**) High magnification view of the field indicated by a red square in (**E**), showing SOX2 (red), OCT4 (cyan) and NANOG (green) fluorescence channels. (**G**, **H**) Quantitative RT-PCR of *Sox3*, *Otx2*, *Pou3f1* and *Fgf5* total mRNA (**G**) or *Sox3*, *Otx2*, *Pou3f1, Fgf5* and *Eomes* nascent transcript (**H**) expression in $Nanog^{-/-}$; $CAG^{NanogERT2\text{-}IRES\text{-}puro}$ (NERT) EpiLCs treated with pEpiLC medium containing (+) or without (−) tamoxifen for the indicated hours. Expression levels are normalised to TBP (**H**) and the −4OHT control (**G**); values are means ± s.d.; points show values for three independent biological replicates.

 © The Author(s)

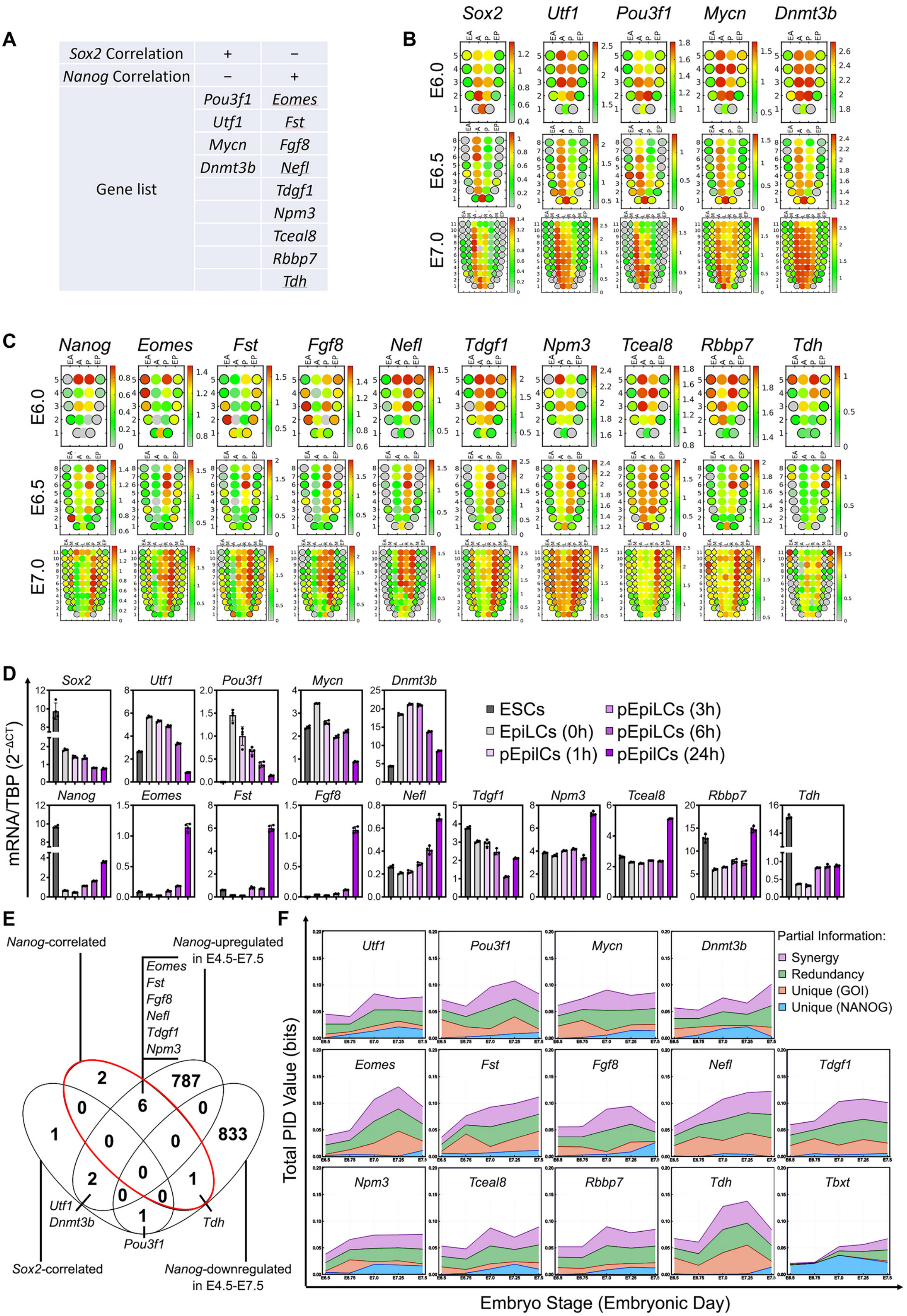
A
Sox2 Correlation + −
Nanog Correlation − +
Gene list
Pou3f1 Eomes
Utf1 Fst
Mycn Fgf8
Dnmt3b Nefl
Tdgf1
Npm3
Tceal8
Rbbp7
Tdh
B
Sox2 Utf1 Pou3f1 Mycn Dnmt3b
E6.0
E6.5
E7.0
C
Nanog Eomes Fst Fgf8 Nefl Tdgf1 Npm3 Tceal8 Rbbp7 Tdh
E6.0
E6.5
E7.0
D
Sox2 Utf1 Pou3f1 Mycn Dnmt3b
Nanog Eomes Fst Fgf8 Nefl Tdgf1 Npm3 Tceal8 Rbbp7 Tdh
mRNA/TBP (2−ΔCT)
ESCs
EpiLCs (0h)
pEpilCs (1h)
pEpiLCs (3h)
pEpiLCs (6h)
pEpilCs (24h)
E
Nanog-correlated
Nanog-upregulated in E4.5-E7.5
Eomes
Fst
Fgf8
Nefl
Tdgf1
Npm3
2
787
0
6
0
1
0
0
833
2
0
1
0
0
1
Utf1
Dnmt3b
Pou3f1
Tdh
Sox2-correlated
Nanog-downregulated in E4.5-E7.5
F
Utf1 Pou3f1 Mycn Dnmt3b
Eomes Fst Fgf8 Nefl Tdgf1
Npm3 Tceal8 Rbbp7 Tdh Tbxt
Partial Information:
Synergy
Redundancy
Unique (GOI)
Unique (NANOG)
Total PID Value (bits)
Embryo Stage (Embryonic Day)

Figure EV8. Supporting Fig. 4.

(**A**) Genes that show consistent opposite correlation between *Sox2* and *Nanog* across more than 1 stage of mouse embryos from E6.5 to E7.5; see also Dataset EV2 Genes that are positively correlated with *Sox2* and negatively with *Nanog* are denoted *Sox2*-correlated genes. Conversely, the opposite set of genes are *Nanog*-correlated genes. (**B**, **C**) Spatial transcriptomic 'corn' plots (of *Sox2* and *Sox2*-correlated genes (**B**) or *Nanog* and *Nanog*-correlated genes (**C**) from E6.0 to E7.0. Each dot represents the laser-captured microdissected section of the mouse embryo. Red is high expression and green is undetected expression. Corn plots were generated using the scGastrulation web portal as described in (Wang et al, 2023). (**D**) Quantitative RT-PCR of *Sox2*, *Nanog* and their respective-correlated genes in 2i/LIF ESCs, EpiLCs and EpiLCs cultured in pEpiLC condition for the indicated hours. Values are normalised to *Tbp* and presented as $2^{-\Delta CT}$. See Fig. 4B for expression normalised to EpiLCs. (**E**) Intersection between Nanog- or Sox2-correlated genes, and genes that change significantly upon Nanog overexpression between E4.5-E7.5 (Tiana et al, 2022) (F) Stacked plots of total partial information decomposition (PID) for predicting Sox2 expression levels in mouse embryos from E6.5 to E7.5. PID values that is contributed uniquely by NANOG (blue) or the indicated *Nanog*-correlated gene-of-interest (GOI, orange) is shown along with redundant (green) or synergistic (magenta) information conferred by NANOG and the indicated GOI for each embryo stage.

 © The Author(s)

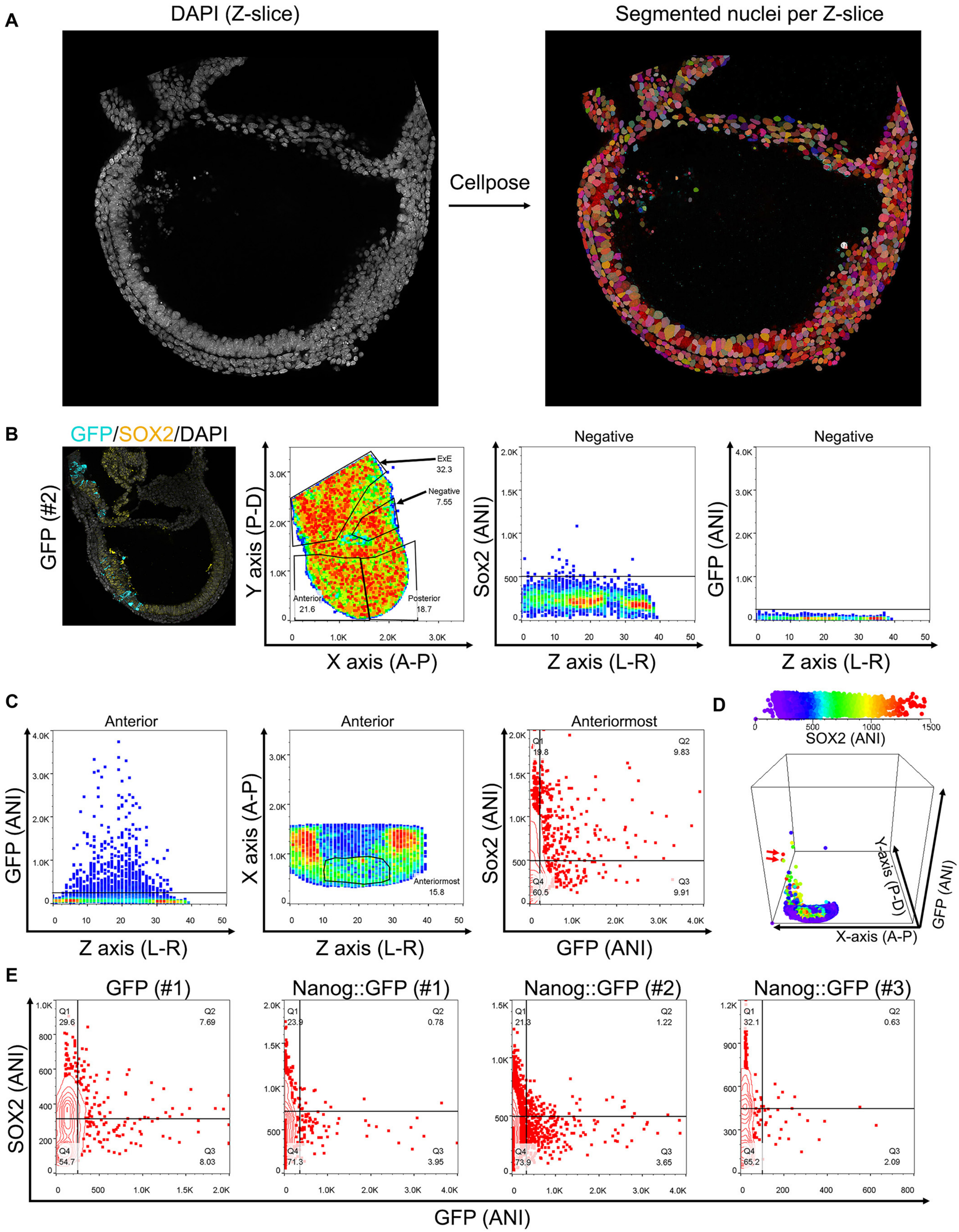
A
DAPI (Z-slice)
Cellpose
Segmented nuclei per Z-slice
B
GFP/SOX2/DAPI
GFP (#2)
Y axis (P-D)
X axis (A-P)
ExE
32.3
Negative
7.55
Anterior
21.6
Posterior
18.7
Negative
Sox2 (ANI)
Z axis (L-R)
Negative
GFP (ANI)
Z axis (L-R)
C
Anterior
GFP (ANI)
Z axis (L-R)
Anterior
X axis (A-P)
Anteriormost
15.8
Z axis (L-R)
Anteriormost
Sox2 (ANI)
Q1
19.8
Q2
9.83
Q4
60.5
Q3
9.91
GFP (ANI)
D
SOX2 (ANI)
Y-axis (P-D)
GFP (ANI)
X-axis (A-P)
E
GFP (#1)
Q1
29.6
Q2
7.69
Q4
54.7
Q3
8.03
Nanog::GFP (#1)
Q1
23.9
Q2
0.78
Q4
71.3
Q3
3.95
Nanog::GFP (#2)
Q1
21.3
Q2
1.22
Q4
73.9
Q3
3.65
Nanog::GFP (#3)
Q1
32.1
Q2
0.63
Q4
65.2
Q3
2.09
SOX2 (ANI)
GFP (ANI)

Figure EV9. Supporting Fig. 5.

(**A**) A representative DAPI-stained Z-slice in a confocal stack was segmented using a machine-learnt model Cellpose to generate segmented nuclear area per each Z-slice in the stack. (**B–D**) Example of workflow to extract Sox2 expression in Nanog-GFP or control GFP electroporated embryos with their neighbours using control GFP embryo 2. (**B**) A representative Z-slice near the midline of an electroporated embryo showing the expression of SOX2 (yellow), GFP (cyan), overlaid on DAPI (grey). The segmented nuclei of this embryo are plotted with its X/Y coordinates, representing the anteroposterior (A–P) and proximodistal (P-D) axes, respectively. Gates were drawn to extract the segmented nuclei in the anterior, posterior, ExE and an internal negative region. SOX2 and GFP ANI thresholds were determined using the negative region along the Z axis (corresponding to the left-right axis) to ensure the threshold captures any diffraction problems due to the thickness of the sample. (**C**) Segmented nuclei with high GFP ANI values were observed in the centre of the Z axis indicating anterior midline electroporation. The electroporated region is extracted to ensure that the SOX2 expression levels are comparable between GFP+ and GFP− cells. The relationship between SOX2 ANI and GFP ANI per segmented nucleus in this anteriormost region is represented in a 10% contour plot showing all events. (**D**) A 3D representation of the segmented nuclei in the embryonic (i.e. anterior + posterior) region plotted with GFP ANI in the vertical axis. The colour of each dot represents the detected Sox2 ANI value (top colour scale) to show that high Sox2 expression is permitted in high GFP-expressing cells without NANOG overexpression. (**E**) Similar contour plots showing the relationship between SOX2 and GFP ANI in another embryo electroporated with *CAG*-GFP in comparison to three independently electroporated embryos with *CAG*-Nanog::GFP.

© The Author(s)

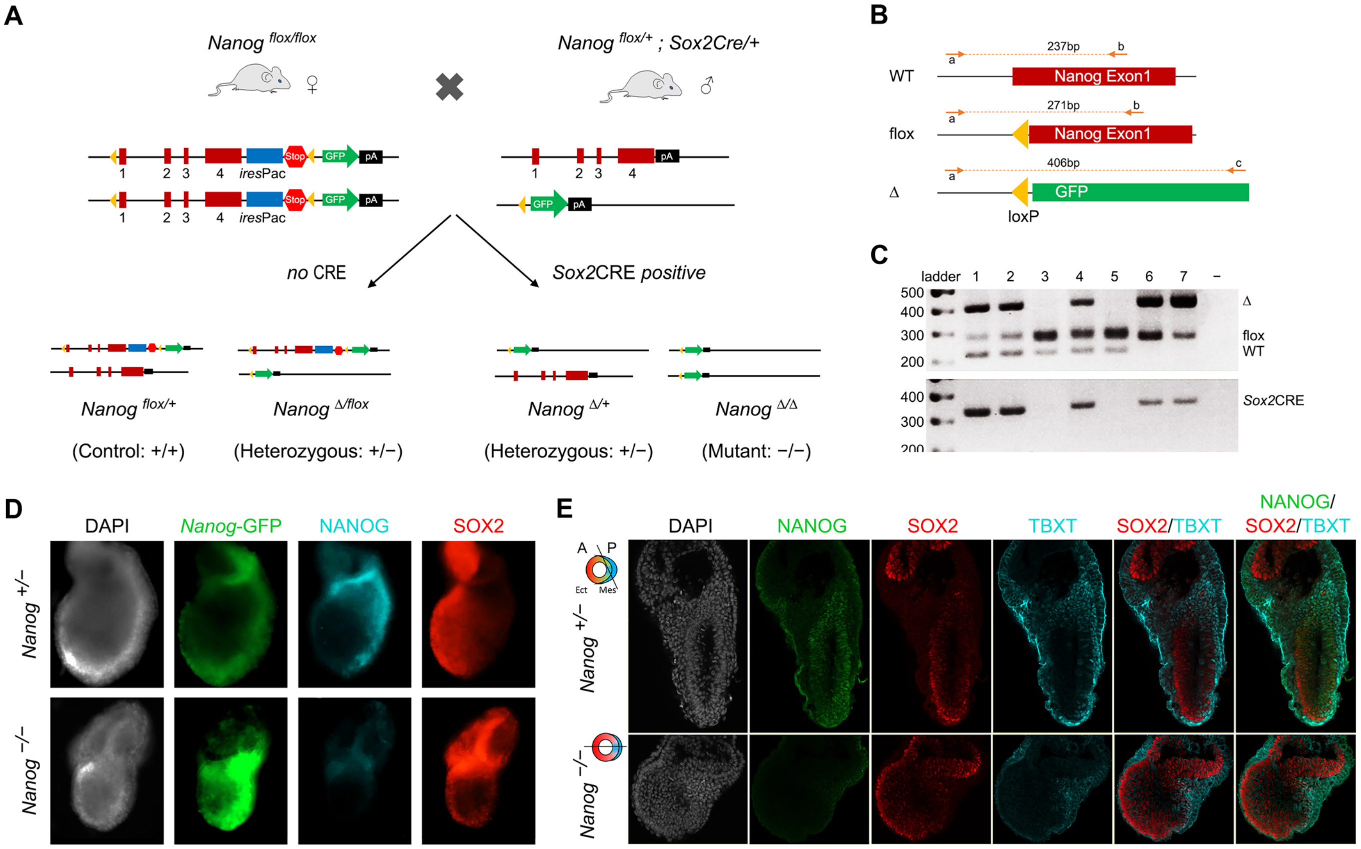

Figure EV10. Supporting Fig. 5.

(**A**) Schematic of *Nanog*-conditional knockout by *Sox2*CRE. *Nanog*$^{flox/flox}$ female mice were crossed with *Nanog*$^{flox/+}$; *Sox2*$^{Cre/+}$ male mice. As *Sox2*CRE is heterozygous, one in four offspring will be *Nanog* conditional knockout (*Nanog*$^{\Delta/\Delta}$). (**B**) Genotyping strategy. Three primers, one forward primer (a) and two reverse primer (b, c) were used. “bp” denotes base pair. (**C**) Genotyping results of embryos derived from *Nanog*$^{flox/flox}$ females crossed with *Nanog*$^{flox/+}$; *Sox2*$^{Cre/+}$ male mice. The extraembryonic regions were lysed for genotyping. Lanes 6 and 7, which have no “WT” band but have flox, Δ and *Sox2*CRE bands, correspond to homozygous *Nanog*-conditional null embryos (*Nanog*$^{\Delta/\Delta}$). (**D**) *Nanog*-GFP (green) fluorescence, NANOG (cyan) and SOX2 (red) immunofluorescence in *Nanog*$^{+/-}$ and *Nanog*$^{-/-}$ embryos. The *Nanog*-GFP excised allele (details in **B**) is still responsive to signals restricting *Nanog*-GFP to the posterior region. (**E**) Representative longitudinal optical sections of *Nanog*$^{+/-}$ and *Nanog*$^{-/-}$ embryos. Schematic shows approximate plane of section on a notional transverse section of each embryo. NANOG (green), SOX2 (red), and TBXT (cyan) immunofluorescence showing TBXT is correctly localised to the primitive streak in SOX2+ cells.

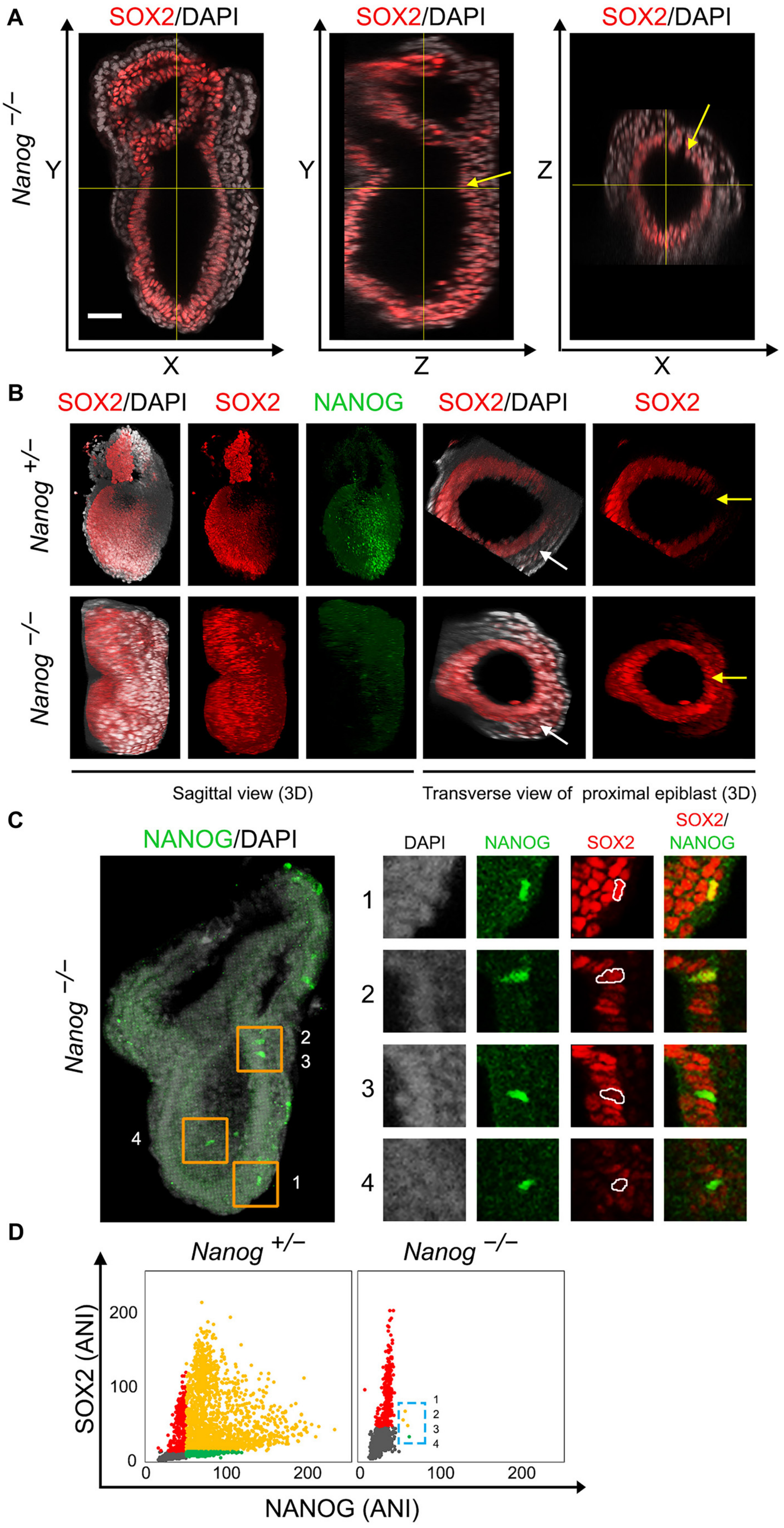
A
SOX2/DAPI
SOX2/DAPI
SOX2/DAPI
Nanog −/−
Y
X
Y
Z
Z
X
B
SOX2/DAPI
SOX2
NANOG
SOX2/DAPI
SOX2
Nanog +/−
Nanog −/−
Sagittal view (3D)
Transverse view of proximal epiblast (3D)
C
NANOG/DAPI
Nanog −/−
DAPI
NANOG
SOX2
SOX2/NANOG
1
2
3
4
D
Nanog +/−
Nanog −/−
SOX2 (ANI)
NANOG (ANI)
0
100
200

 © The Author(s)

Figure EV11. Supporting Fig. 5.

(**A**) Orthogonal view of a *Nanog*$^{-/-}$ embryo. Left, XY projection. Middle, YZ projection, approximating a sagittal section with anterior to the left. Righthand image, XZ projection, shows the posterior epiblast expressing SOX2 (yellow arrow). SOX2 = red; DAPI = grey; scale bar = 40 µm. (**B**) 3D rendering showing sagittal (left) and transverse (right) views of the *Nanog*$^{-/-}$ embryo in (**A**) along with a *Nanog*$^{+/-}$ embryo from the same dataset. The transverse view was generated from an equivalent cropped region of the proximal embryonic region to that shown in Fig. 5C. *Nanog*$^{+/-}$ embryos showed no SOX2 posteriorly, whereas SOX2 was detected in the posterior epiblast (yellow arrow) and in the ingressed mesoderm (white arrow) of *Nanog*$^{-/-}$ embryos. (**C**) Left, maximum Z projection of a segment of a second *Nanog*$^{-/-}$ embryo containing the location of the detected Nanog-positive cells (orange boxes, numbered). Right, each NANOG+ cell is shown overlaid on SOX2 expression. (**D**) Quantitation of SOX2 (red) and NANOG (green) ANI in a representative *Nanog*$^{+/-}$ or *Nanog*$^{-/-}$ embryo. A total of 3 *Nanog*$^{+/-}$ and 6 *Nanog*$^{-/-}$ embryos were analysed. Thresholds were gated using an internal negative region. The 4 detected Nanog-positive cells (blue box, numbered in order of SOX2 ANI level) had low or negative SOX2 ANI.

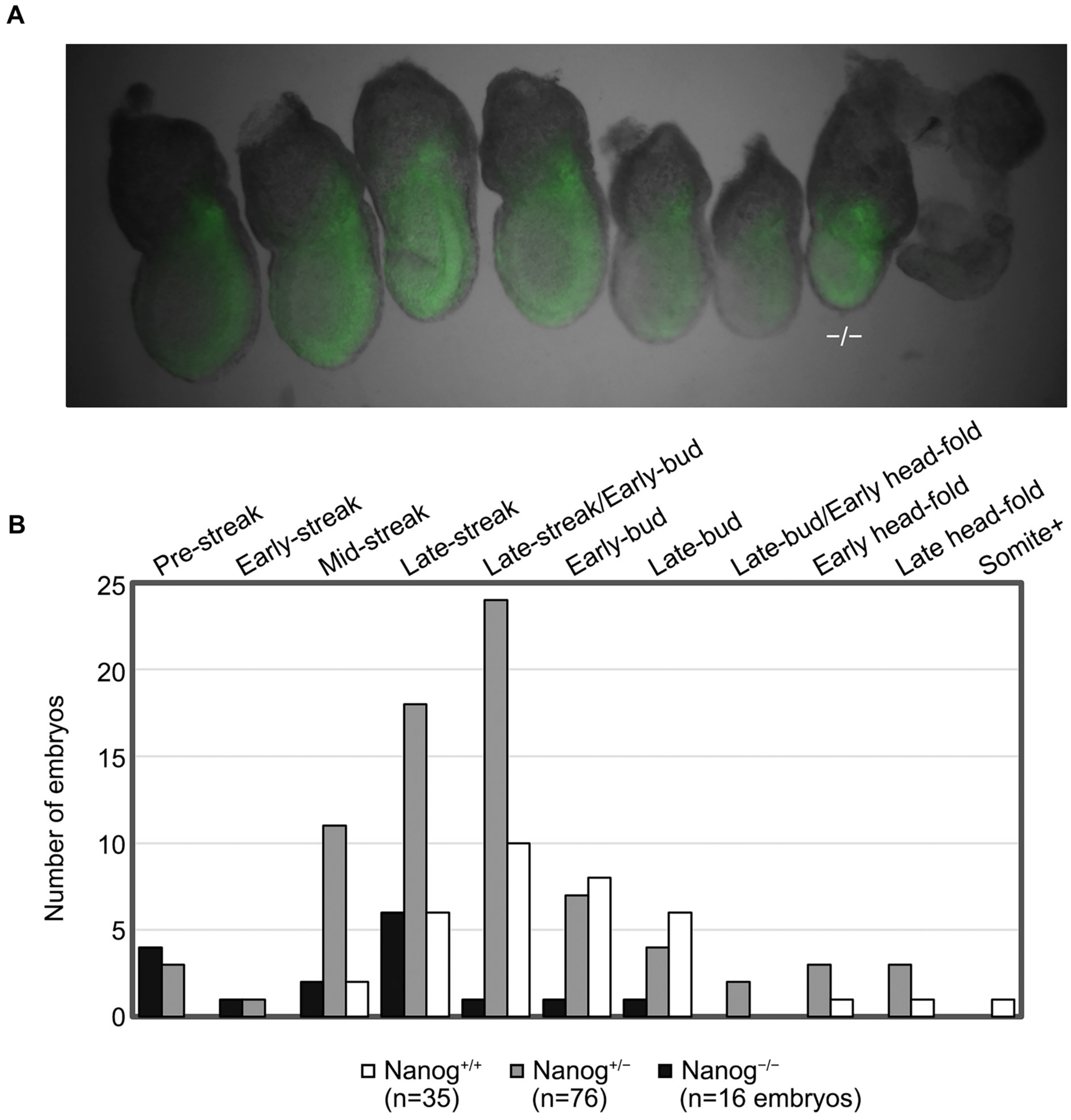

Figure EV12. Supporting Fig. 5.

(**A**) Composite bright-field and *Nanog*-GFP fluorescence images of freshly dissected embryos at E7.5. *Nanog* genotyping (see Fig. EV11A for details) results are indicated below each embryo. (**B**) *Nanog*$^{+/+}$ (white bars), *Nanog*$^{+/-}$ (grey bars) and *Nanog*$^{-/-}$ KO (black bars) embryos were scored by the morphological landmarks of the Downs and Davies staging prior further analysis. The total numbers of embryos analysed across all experimental replicates for each genotype are indicated.

 © The Author(s)
